# Genome-scale metabolic rewiring improves titers rates and yields of the non-native product indigoidine at scale

**DOI:** 10.1038/s41467-020-19171-4

**Published:** 2020-10-23

**Authors:** Deepanwita Banerjee, Thomas Eng, Andrew K. Lau, Yusuke Sasaki, Brenda Wang, Yan Chen, Jan-Philip Prahl, Vasanth R. Singan, Robin A. Herbert, Yuzhong Liu, Deepti Tanjore, Christopher J. Petzold, Jay D. Keasling, Aindrila Mukhopadhyay

**Affiliations:** 1 Joint BioEnergy Institute, Lawrence Berkeley National Laboratory, Emeryville, CA 94608 USA; 2 Biological Systems and Engineering Division, Lawrence Berkeley National Laboratory, Berkeley, CA 94720 USA; 3 Advanced Biofuel and Bioproduct Process Development Unit, Lawrence Berkeley National Laboratory, Emeryville, CA 94608 USA; 4 Joint Genome Institute, Lawrence Berkeley National Laboratory, Berkeley, CA 94720 USA; 5 QB3 Institute, University of California-Berkeley, 5885 Hollis Street, 4th Floor, Emeryville, CA 94608 USA; 6 Department of Chemical & Biomolecular Engineering, University of California, Berkeley, CA 94720 USA; 7 Department of Bioengineering, University of California, Berkeley, CA 94720 USA; 8 Novo Nordisk Foundation Center for Biosustainability, Technical University Denmark, 2970 Horsholm, Denmark; 9 Synthetic Biochemistry Center, Institute for Synthetic Biology, Shenzhen Institutes for Advanced Technologies, Shenzhen, China; 10 Environmental Genomics and Systems Biology Division, Lawrence Berkeley National Laboratory, Berkeley, CA 94720 USA

**Keywords:** Metabolic engineering, Applied microbiology, Synthetic biology

## Abstract

High titer, rate, yield (TRY), and scalability are challenging metrics to achieve due to trade-offs between carbon use for growth and production. To achieve these metrics, we take the minimal cut set (MCS) approach that predicts metabolic reactions for elimination to couple metabolite production strongly with growth. We compute MCS solution-sets for a non-native product indigoidine, a sustainable pigment, in *Pseudomonas putida* KT2440, an emerging industrial microbe. From the 63 solution-sets, our omics guided process identifies one experimentally feasible solution requiring 14 simultaneous reaction interventions. We implement a total of 14 genes knockdowns using multiplex-CRISPRi. MCS-based solution shifts production from stationary to exponential phase. We achieve 25.6 g/L, 0.22 g/l/h, and ~50% maximum theoretical yield (0.33 g indigoidine/g glucose). These phenotypes are maintained from batch to fed-batch mode, and across scales (100-ml shake flasks, 250-ml ambr®, and 2-L bioreactors).

## Abstract (web summary)

The trade-off between growth and production affects the application of engineered microbes. Here, the authors take the minimal cut set approach to predict metabolic reactions for elimination to couple metabolite production strongly with growth and achieve high production of indigoidine in *Pseudomonas putida*.

## Introduction

Synthetic biology approaches for heterologous, non-native bioproducts, can provide alternative sustainable routes to a vast number of chemicals ranging from fuels and commodities to fine chemicals. Heterologous production has been demonstrated for many desirable compounds and in a wide variety of microbial hosts^1,2^. Yet, even the most well-designed heterologous pathway requires considerable additional work to reach the titers, rate and yield (TRY) necessary for the adoption of these systems by industry^3,4^. In addition, the production parameters of a strain at lab-scale are often not predictive of its performance and robustness when cultivated in different modes or at larger scales. Consequently, only a small fraction of such bioproduction strains have been successfully scaled and deployed^2^. In contrast, many native microbial processes show high productivity and reliability at scale and represent the most prominent examples of successful high-volume bioproduction. Examples include the generation of ethanol^5^ and organic acids^6,7^ during fermentation where production of these metabolites are required for carbon utilization during fermentative growth. Recently, a minimal cut set (MCS)-based approach^8^ showed that theoretically, production of a majority of metabolites can be strongly coupled to growth via elimination of a minimal set of metabolic reactions. Strong growth coupling^8,9^ is defined as metabolic rewiring, which demands production of the target metabolite (e.g., to generate ATP for non-growth-associated maintenance processes) even when cells do not grow. Metabolic flux towards unwanted side-products is minimized, and metabolite production is feasible even when cell growth is sub-optimal or negligible. In this study we examine if coupling production of a heterologous product to microbial growth is possible, and if such dependence could lead to high TRY and the ability to maintain production parameters across different growth modes and scales.

We use indigoidine^10^, a non-ribosomal peptide, as the heterologous product to prototype our approach. Indigoidine is a viable alternative for colorants in the dye, ink, and pigment industry^11^. Development of a robust production system for this compound stands to have an immediate benefit as a sustainable dye in the garment industry where the use of petrochemical derived dyes contribute to its negative impact on the environment^12–14^. We implemented this system in *Pseudomonas putida* KT2440 (an industrially relevant production host^15^), leveraging the availability of the iJN1462 genome scale metabolic model (GSMM) for *P. putida* KT2440^16^.

Here, we use the minimal cut set (MCS)-based approach^8^ to compute intervention strategies that enforce strong growth coupled product formation. We combine these analyses with publicly available -omics data^17,18^ to exclude essential genes from editing. The corresponding set of gene loci are repressed using multiplex CRISPR interference (CRISPRi), which we optimize for use in *P. putida* KT2440. Our implementation results in a highly edited strain that, in a single iteration of strain engineering, achieves close to 50% max theoretical yield of indigoidine in *P. putida* KT2440 and TRY characteristics that maintain fidelity from laboratory to industrially relevant scales.

## Results

### Genome scale evaluation of *P. putida* for strong coupling

**Fig. 1:**
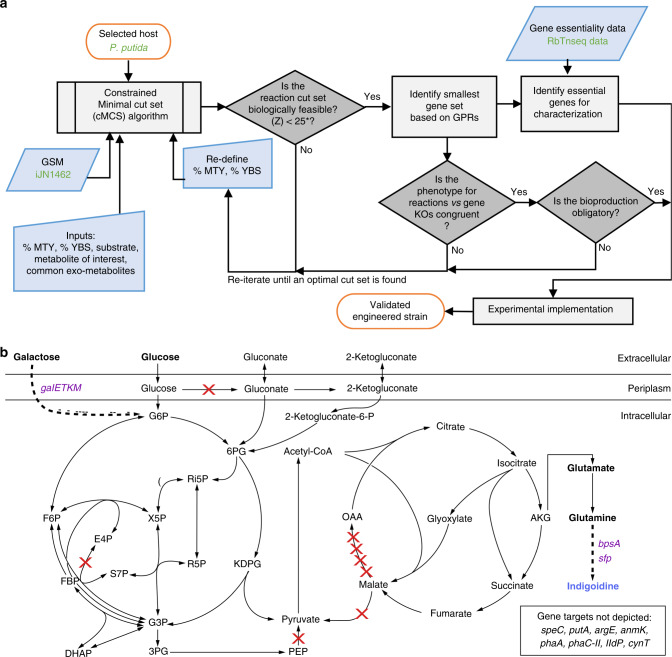
Computationally guided predictions for metabolic rewiring in *P. putida*. **a** Modeling and engineering workflow diagram. This approach can potentially be extended to any carbon source, host and/or metabolite. Input specific to this specific host/final product work is marked in green font. **b** The central metabolism of *P. putida* engineered to produce indigoidine from either glucose or galactose. Heterologous genes are indicated in purple text. Indigoidine is derived from the TCA intermediate α-ketoglutarate (AKG) via two molecules of glutamine. The genes targeted in *P. putida* central metabolism for knockdown by dCpf1/CRISPRi are indicated with red X marks. Additional gene targets outside of *P. putida* central metabolism are indicated in the box on the bottom right. A total of 14 genes were targeted for CRISPR interference excluding *mqo-I* and *cynT*, as the latter are essential by genome-wide transposon mutagenesis (RB-TnSeq). Abbreviations include Genome-scale Model (GSM), maximum theoretical yield (MTY), size of reaction cut set (Z), Gene–protein-reaction relationships (GPRs), knockout (KO), biomass yield (YBS), glucose-6-phosphate (G6P), fructose-6-phosphate (F6P), fructose-1,6-biphosphate (FBP), 6-phosphogluconate (6PG), 2-keto-3-deoxy-6-phosphogluconate (KDPG), ribulose-5-phosphate (Ri5P), ribose-5-phosphate (R5P), xylulose-5-phosphate (X5P), sedoheptulose-7-phosphate (S7P), erythrose-4-phosphate (E4P), glyceraldehyde-3-phosphate (G3P), glycerate-3-phosphate (3PG), dihydroxyacetone phosphate (DHAP), phosphoenolpyruvate (PEP) and oxaloacetate (OAA). Refer to Supplementary Fig. 1 and Supplementary Data 1 for more information.

To develop the product coupling approach (Fig. 1a), we first identified all potential metabolites represented in *P. putida* iJN1462^16^ model that can be made essential for growth. For this analysis we used the MCS algorithm^8^ that identified minimal sets of reactions (cut sets), the elimination of which would enforce strong growth coupled production of a given metabolite (see “Methods”). Our initial analysis revealed that for *P. putida* around 99% of the producible metabolites accounted for in the genome scale model had the potential for strong growth coupling. This potential growth coupling for all metabolites is consistent with reported calculations demonstrated in other hosts^8^ (Supplementary Table 1). However, the percentage of potential strong growth coupled metabolites was reduced from 99 to 45% when a higher minimum product yield was demanded. This metabolite-level analysis provided reaction information, but needed to be resolved into specific enzymatic reactions to implement experimentally.

Next, we evaluated if an obligatory regime is possible for the heterologous product indigoidine, accounting for other known biological limitations. The process for determining possible gene targets and assessing implementability is diagrammed in Fig. 1. We began by adding an in silico reaction for indigoidine, to the genome scale metabolic model iJN1462^16^. This reaction represents the biosynthesis of indigoidine from glutamine and includes ATP and flavin mononucleotide (FMN) as cofactors. The maximum theoretical yield (MTY) for glutamine and indigoidine was calculated to be 1.141 mol/mol and 0.537 mol/mol, respectively, from glucose as the carbon source (Table 1). The MTY for glutamine in *P. putida* was high relative to several other hosts (Supplementary Table 2). As this method accounts for the other physiological processes competing for resources, a MTY derived from a genome scale model provided a more accurate assessment compared to simpler methods, as is common in the field^19,20^. We chose glutamine, the direct precursor to indigoidine, as the growth coupled final product so as to bypass other essential cellular processes, which also use FMN as a cofactor^21^. The minimum theoretical product yield of glutamine was set at 10, 50, 70, 80, and 85% MTY to derive the reactions that would require knockout or knockdown for strong growth coupling. To determine which gene loci represent the reactions from the predicted constrained minimal cut set (cMCS), we then needed to extract the gene–protein-reaction relationships (GPRs) for each of the reactions embedded in the GSMM to translate the selected metabolic reactions into specific genes and assess their role in other functions. We eliminated potential cut sets that targeted genes coding for multi-functional proteins, as we sought to limit additional metabolic perturbations that could confound our analysis. A total of 882 reactions were excluded from consideration. These included spontaneous reactions, exchanges, transporters, some core essential reactions such as ATP maintenance, ATP synthesis reactions, reactions associated with the TOL plasmid genes and reactions that were not assigned a GPR. A total of 826 reactions out of 2928 were not associated with any genes. Of these 826 reactions, 740 reactions are either exchanges, transporters, biomass reaction, ATP maintenance, demand or sink reactions. Only 86 (10%) are true cytosolic reactions that might be associated with unidentified GPR. Their inclusion in the solution under such constraints would assist in identifying unknown GPRs and hold promise for further investigation. Of the 2030 reactions in iJN1462 that are associated with genes, only 60% have a single gene associated with them. If a metabolic reaction was catalyzed by more than one gene product (genes coding for isozymes or multi-subunit enzymes), we included both genes for inactivation. From this workflow, we analyzed 63 cMCS in total but only one feasible cMCS emerged with the predicted potential for high indigoidine titer (Supplementary Table 3). Using a minimum threshold of 80% MTY indigoidine and 10% of maximum biomass yield our feasible cut set targeted 14 metabolic reactions. Eight of these 14 reactions are present in central metabolism and when mapped to their corresponding genes and gene products, represent 16 single-copy genes dispersed throughout the genome (Fig. 1b and Supplementary Data 1). A full depiction of all reactions targeted for inhibition is described in Supplementary Fig. 1.

**Table 1:** Maximum theoretical yield of glutamine and indigoidine from two different substrates glucose and galactose with respect to stoichiometry and redox balance in *P. putida*.

Product	mol product/mol glucose	g product/g glucose	mol product/mol galactose	g product/g galactose
α-ketoglutarate	1.320	1.07	1.366	1.11
Glutamine	1.141	0.93	1.181	0.96
Indigoidine	0.537	0.74	0.556	0.77

We then sought to confirm if the set of specific genes (rather than enzymatic reactions) for intervention was metabolically sound. For this we used flux balance analysis (FBA) and flux variability analysis (FVA) to confirm that the 16-gene cMCS strategy resulted in obligatory glutamine production. Using our constructed MCS algorithm-based platform (Fig. 1a), we set the parameters to explore potential product-obligatory strategies to enhance the production of indigoidine in *P. putida* when glucose was fed as the sole carbon source. This 16-gene set provided for glutamine was then extended to assess production paired growth for indigoidine. FBA analyses confirmed that growth using glucose could support indigoidine production at 90% theoretical yield (0.48 mol/mol or 0.66 g/g of glucose).

Since the MCS-based approach requires the delineation of specific growth conditions, such as starting carbon source, we examined if the gene cut set with glucose as a substrate could maintain product pairing with other known native carbon substrates for *P. putida*, such as *para*-coumarate and lysine^17,22^. These substrates are important carbon streams that could be utilized from lignocellulosic biomass hydrolysates^23,24^. FBA with these alternate carbon sources (i.e., lysine, *para-*coumarate) indicated that a strain engineered using the 16-gene cMCS strategy for the glucose would fail to produce glutamine (Supplementary Table 4). This gene targeting set (Supplementary Data 1) should also result in the desired production obligatory growth using galactose (a hemicellulosic-derived sugar) as a carbon source, confirmed using FBA, because it shares the same downstream catabolism as glucose (Fig. 1b). While *P. putida* cannot natively consume galactose, the galactose catabolic pathway has been well characterized in *E. coli*^25,26^.

### Building the multi-edit engineered strain

Testing the predictions for indigoidine production required an extensively engineered strain. First we built the *P. putida* indigoidine production platform by genomically integrating a heterologous production pathway composed of *sfp* and *bpsA*. BpsA is a non-ribosomal peptide synthetase (NRPS)^27^ from *Streptomyces lavendulae* that catalyzes indigoidine formation from two molecules of glutamine in an ATP-dependent manner^10^. Activation of BpsA requires a post-translational pantetheinylation conferred by a promiscuous Sfp from *Bacillus subtilis*^28^. The genomically integrated production strain harboring a plasmid-borne *dCpf1* and non-targeting genomic RNA (gRNA) serves as the control production strain. The basal production of indigoidine in *P. putida* is 2.3 g/L indigoidine from 10 g/L glucose after 24 h (Supplementary Fig. 2a). The bulk of production occurred in stationary phase, ~12 h after carbon depletion. Production in late exponential or stationary phase is typical for several products in *P. putida*^29,30^. To test the use of galactose as a carbon source, we also engineered a galactose utilization strain via genomic integration of a *galETKM* operon^25,26^ and here production of indigoidine was negligible (Supplementary Fig. 2b). Optimizing the carbon/nitrogen ratio yielded only modest improvements to indigoidine production for both glucose and ammonium sulfate (Supplementary Fig. 2c–e).

Before designing experimental strategies for building the multi-gene edited strain we needed to remove known inconsistencies from the iJN1462 model^16^. We assessed if our predicted gene set contained essential genes. We observed that the iJN1462 model has an incomplete assessment of essential genes; we manually annotated genes as essential or dispensable using gene essentiality data generated from a *P. putida* KT2440 barcoded fitness library (RB-TnSeq)^18^ (Supplementary Data 2). Out of the 16 genes identified for knockdown, two genes were excluded because they are essential for viability by RB-TnSeq analysis. By eliminating essential genes from the targeted gene set, we hypothesized that the predicted metabolic rewiring most accurately represents a product/substrate pairing rather than growth coupling, as the smaller targeted gene set does not make a bounded prediction on how growth rate could be altered unless additional constraints on growth is implemented.

To efficiently overcome technical limitations required to make 14 gene edits, we implemented a multiplex dCpf1/CRISPRi targeting strategy. We drew on our understanding of repetitive element instability^31,32^ to minimize use of repeated DNA sequences to limit gRNA array loss. While other reports have indicated technical challenges constructing multiplex gRNA arrays^33^, native arrays exist in nature and synthetic arrays have been generated (including those of native CRISPR arrays)^34,35^. An endonuclease-deficient class II CRISPR-Cas enzyme, FnCpf1^36^, was chosen over *Streptococcus pasteurianus* dCas9 as the Cpf1 crRNA is only 19 bp in size, compared to the corresponding crRNA (gRNA scaffold sequence) from Cas9, which is 76 bp^37^. Each gRNA was driven by a different *P. putida* tRNA ligase promoter/terminator pair, and dCpf1 was placed under the control of the *lacUV5* promoter. Minimal 100-bp promoter sequences from native tRNA ligases were sufficient to express *mCherry* fluorescent protein, confirming that heterologous messenger RNA (mRNA) transcripts for gRNAs would be generated (Supplementary Fig. 2f).

**Fig. 2:**
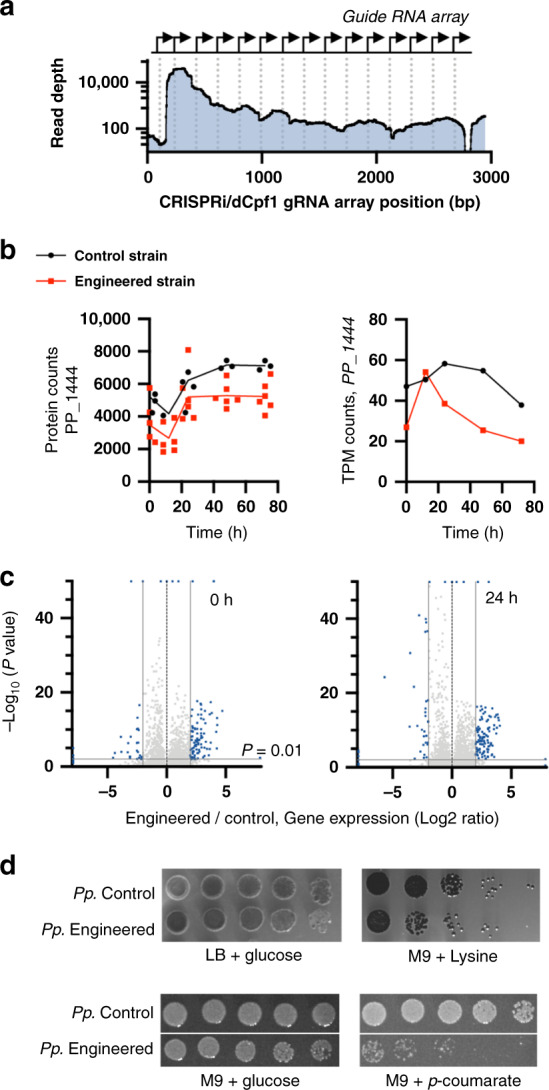
Characterization of the multi-gene engineered strain via RNAseq and proteomics. **a**–**c** *P. putida* harboring a genomically integrated indigoidine expression cassette and either an empty vector (control strain) or a dCpf1/CRISPRi targeting array examined for gene knockdown efficiency. **a** RNAseq analysis of plasmid-borne gRNA array targeting 14 genes (Fig. 1a, and Supplementary Data 1) in *P. putida*. **b** Knockdown efficiency of a representative gene locus PP_1444 (*gcd*) targeted for inhibition over a 72-h time course. RNA expression levels (right hand panel) were validated with targeted proteomic analysis (left hand panel). Proteomic samples were analyzed with *n* = 3 for control samples and *n* = 6 for the engineered strain. For the RNAseq analysis for the control sample and *n* = 2, *n* = 4 for the engineered strain. For the proteomics sample, all data points are shown. Transcripts per kilobase million (TPM) counts from a representative RNAseq time course are shown. **c** dCpf1/CRISPR interference causes global RNA expression level changes. Volcano plot of mRNA expression levels compared at *t* = 0 h and *t* = 24 h between multi-gene engineered and control strains. 184 data points (0 h) and 391 data points (24 h) out of 5369 data points are outliers some are displayed on the edge of the axes. The *y*-axis indicates the expression value of log_10_(*q*-value), and the *x*-axis displays the log_2_fold change. The blue dots represent gene expression levels that were significantly different, and the dotted blue line indicates the threshold where *P* = 0.01. *P*-values were calculated using Fisher’s exact test. Gray dots indicate transcripts that were not statistically significant. **d** Validation of carbon source rewiring. Genome-scale modeling predicts that glucose/indigoidine rewiring blocks growth of engineered strains on lysine or *para*-coumarate as a carbon source. A representative set of plates is shown from three biologically independent experiments. Source data are provided as a Source Data file.

Experimental validation revealed that the 14 gene simultaneous knockout resulted in a knockdown of 9 out of the 14 genes. Successful deployment of the multiplex dCpf1/CRISPRi should result in a decrease in mRNA expression levels (and protein abundance) of the genes targeted with CRISPR interference. We used RNAseq analysis to examine the engineered strain, and compared normalized RNA expression levels to the control strain (Fig. 2a–c). RNA expression levels were sampled over the duration of a 72-h time course. Expression of all 14 gRNAs was detected by this analysis (Fig. 2a). The multiplexed Cpf1 gRNAs in this array did not efficiently terminate with endogenous terminator sequences and generated chimeric mRNAs. Nonetheless, nine of the fourteen targeted gene loci exhibited decreased mRNA expression levels, and at best showed a 50% decrease (Fig. 2b and Supplementary Fig. 3). No commonalities for gRNA knockdown efficiency (such as position in ORF/promoter or targeting the sense vs. antisense strand) were discernible **(**Supplementary Data 1**)**. Indirect changes in gene expression were detected (Fig. 2c), consistent with a report using a smaller number of multiplex CRISPRi targets in *E. coli*^33^. Higher normalized RNAseq counts for gRNAs did not strongly correlate with targeted genes, which were more efficiently inhibited by either RNAseq or proteomic analysis. We were able to confirm that the protein abundance for ten of the targeted genes were also reduced using liquid chromatography with tandem mass spectrometry (LC-MS/MS; Fig. 2b and Supplementary Fig. 3). Multiplex CRISPRi-mediated gene knockdown of these product/substrate pairing targets did not measurably change growth rates when transformed into either the indigoidine production strain or wild type (Supplementary Fig. 2g, h). These results indicate that while the multiplex dCpf1/CRISPRi knockdown strategy only led to modest reductions in protein levels, it was consistent with another report^38^ on knockdown efficacy using dCas9/CRISPRi for this organism.

A consequence of pairing production to the catabolism of specific carbon sources is a prediction that other carbon sources can no longer be metabolized (Supplementary Table 4). We tested this prediction experimentally and observed that engineered strains for product/substrate pairing showed reduced growth when using either lysine or *para-*coumarate as the sole carbon source, in agreement with the modeling (Fig. 2d). This result indicates that while we had not blocked flux for the full set of required reactions, we were still able to obtain measurable changes to cell physiology.

### Characterizing the multi-gene engineered production strain

We next asked if the multi-gene engineered strain was sufficient to yield phenotypic growth consistent with product/substrate pairing or possibly true growth coupling. It was possible that the degree of knockdown was sufficient to observe high TRY for our desired product since higher glutamine yields, to support growth, should result in more indigoidine. The production of indigoidine would shift from stationary phase to exponential phase, as the metabolism of glucose catabolism and glutamine production are paired. Finally, these phenotypes should maintain fidelity across a range of growth modes and scales.

**Fig. 3:**
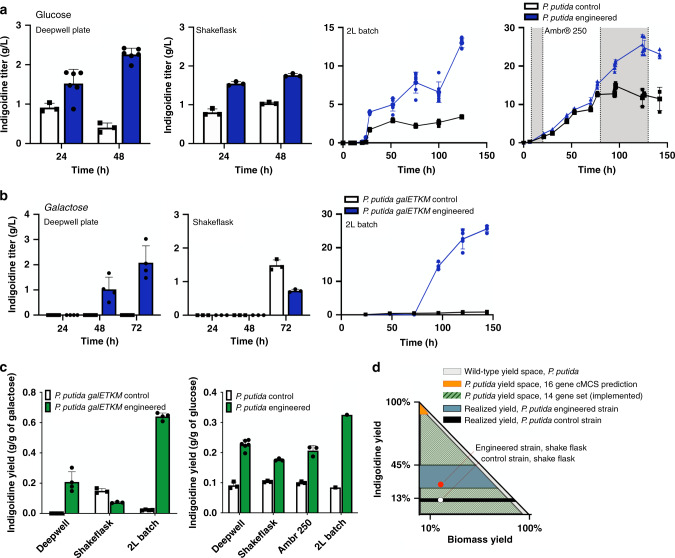
The product substrate pairing approach can improve titer rate and yield across two carbon sources. **a**, **b** Analysis of *P. putida galETKM* multi-gene engineered strains and a control strain (*P. putida galETKM*, empty vector plasmid) for production of indigoidine using glucose (**a**) or galactose (**b**) as the sole carbon source in M9 minimal medium. The culture format assessed is indicated above each panel. A fed-batch mode of cultivation was implemented in the ambr® 250 cultivation format. Glucose feeding is indicated by the gray shaded area. Control samples indicated with black outlined bars or black dots. The multi-gene engineered strains are indicated with blue bars or blue dots. **c** Analysis of indigoidine yield across cultivation formats for both glucose-fed and galactose-fed strains. Yield from the control strain is indicated with black bars, and the multi-gene engineered strain is indicated with green bars. For the deep-well plate and shake flask experiments, data are presented as mean ± SD with an overlay of corresponding data points from *n* = 3 independent experiments. When the engineered strain was tested in the deep-well plate format and fed glucose, *n* = 6 independent experiments. For the industrially relevant formats (2 L and ambr250®) data are presented as mean ± SD with an overlay of corresponding data points from biological duplicate and sampled each in technical triplicate. **d** Computed production envelope using genome scale model and constraint-based methods represented as theoretical yields of indigoidine as a function of biomass yields. Possible yield space for wild-type *P. putida* (gray fill), predicted 16-gene cMCS (yellow fill), down-selected and implemented 14 gene set (hashed green fill). The range of observed yield space for either the control (black fill) or engineered strain (teal fill) across different production formats is represented. The realized production yield vs. biomass yield in the shake flask format for engineered and control strain are represented by a red and white dot respectively. The phase shift in production from stationary phase to exponential is not depicted. Source data are provided as a Source Data file.

Indigoidine production was substantially improved in the engineered strain relative to the controls across several laboratory cultivation formats. We tested production using both the native glucose and engineered galactose pathways as carbon sources. Both strains were cultivated with either 10 g/L glucose or galactose, as the same targeted reaction set would function on either carbon source. In a deep-well plate format, we observed that the engineered strain produced nearly threefold more indigoidine than the control strain when fed glucose (Fig. 3a). In a shake flask format, the engineered strain produced 30% more than the control strain. Notably, when cultivated with galactose in the deep-well format, the same engineered strain was able to produce indigoidine in contrast to the galactose utilization control strain, which only formed biomass (Fig. 3b). In these standard laboratory formats where headspace and aeration can contribute to variation^39^ to product titer, the engineered strain showed slightly higher indigoidine titers in the deep-well plate format.

In a 2-L bioreactor, cultivated in a batch-mode with glucose as the carbon source, we observed an improved titer (2.5-fold) of 12.5 g/L indigoidine from 60 g/L glucose. The control production strain produced 5 g/L, and production of the final molecule was realized after glucose was exhausted from the medium. When galactose was fed, the engineered strain also exhibited improved titers of 25.6 g/L of indigoidine from 60 g/L galactose as opposed to the control strain that generated only around 900 mg/L of indigoidine; a 28-fold improvement in production was observed in the engineered strain. Moving to an industrially relevant cultivation format did not impact the final product titer, allowing us to further develop cultivation methods by switching to fed-batch mode.

We realized greater improvements in final product titer as well as improvements in production kinetics in the fed-batch mode using the ambr® 250 system. After administering an initial high-nutrient feed to increase biomass in the reactor, we reduced the feed rate to study indigoidine product formation during exponential phase growth (Fig. 3a, right hand panel, and Supplementary Fig. 4). During this phase, the engineered strain produced indigoidine at a rate of 0.22 g/l/h, while the control strain accumulated no additional product. This observation is consistent with our hypothesis that indigoidine formation would occur during exponential phase due to pairing with glucose. In terms of yield, the engineered strain generated consistently higher production than the control strain when cultivated with glucose (0.2 g/g compared to 0.1 g/g), (Fig. 3c). Altogether all aspects of the phenotypes that were desirable for the engineered strain were found to be true.

## Discussion

This study implements genome scale MCS predictions via CRISPR interference that resulted in a strain where production was paired with growth. Pairing the final desired product with the carbon substrate for growth mimics native obligatory product formation such as ethanol production and results in high productivity at scale. Further, to our knowledge, there are no other reports where the production of a non-native molecule was shifted from stationary phase to exponential phase as a result of strain engineering.

The competition between biomass accumulation and production of the target compound is a well-recognized challenge in biomanufacturing. This trade-off impacts both TRY and scalability. Approaches to address this trade-off range from growth coupling^7,40^ to growth decoupling^41^. Canonical approaches to growth coupling are FBA-based methods such as OptKnock^42^ and are conceptually different from MCS-based methods. Optknock and related methods (RobustKnock, OptORF, etc.) use bi-level optimization to identify gene edits, which increase flux to the target of interest around the solution space that maximizes growth. The MCS algorithm does not maximize growth and is unbiased towards any part of the solution space. Further, the MCS algorithm allows us to set boundaries for minimum demanded production and growth, thus providing strong coupling constrained MCS (cMCS) solution-sets. These optimization strategies make different assumptions on cellular metabolism and have been extensively reviewed elsewhere^42–44^. OptKnock related methods have been used in growth coupled strategies for primary alcohols (1,4-butanediol^45^) and organic acids (succinate^46^, lactate^47^). However, for a large subset of native metabolites, including amino acids (serine, glutamate, glutamine), FBA-based approaches often result in “non unique” solutions, which are optimized for production but not necessarily for growth. In contrast, the recently delineated MCS-based approach^8^ provides strong coupled solutions sets for a large number of metabolites within a reasonable computation time and fit well with the downstream biological constraints for CRISPR/dCpf1 rewiring. A comparison of solution-sets generated with OptKnock (Supplementary Table 5 and Supplementary Table 6), versus that with cMCS (using similar computation time, targetable reactions, number of reactions to be deleted, etc.), did not provide strong coupled strategies although several ‘non unique’ solutions exist. Other studies have described growth coupling as the creation of a driving force such as ATP production or cofactor imbalance, and link the driving force to the desired production pathway^40,48–50^. Driving force coupling is also pathway specific and requires additional strain engineering. Examples include 1-butanol production in *E. coli* using NADH as the driving force^48^ or media supplementation for butanone production in *E. coli* linked to acetate assimilation^40^.

As defined in the introduction, strong growth coupling demands production of the target metabolite (e.g., to generate ATP for non-growth associated maintenance processes) even when cells do not grow. Our approach relies on computation of cMCS to provide targets at the genome-scale level^8,51,52^ but predicts a large number of enzymatic reactions for elimination. To date, these methods have not been validated experimentally for a heterologous gene product. We used FBA to corroborate our optimal cMCS and removed essential genes from targeted gene sets using -omics data to determine the genes that should be targeted for CRISPRi. Our workflow assesses the output from the MCS algorithm on important quality metrics, which helped to in silico eliminate infeasible solutions that would be experimentally invalid. Most recently, the MCS algorithm was extended to integrate GPRs before the cMCS are computed^53^ that would streamline an inefficient step in our workflow. This genome scale approach (Fig. 1) also represents a valuable paradigm for the evaluation of microbial hosts for their production capacity and could significantly reduce the time taken to optimize carbon source conversion to the final product. The appeal of this strategy is that the gene knockdown solution is scale-agnostic; the predicted metabolic rewiring should apply even in the largest bioreactor formats. The cMCS-based multi-gene engineered product/substrate pairing we report here is an implementation of strong growth coupling^8,9^.

While our engineered strains showed many desirable phenotypes, several aspects merit additional discussion. The predicted constrained minimal cut set (cMCS) demands zero flux through these reactions for strong growth coupling. We excluded two genes from the predicted gene set due to their essentiality. Of the remaining gene targets, our RNAseq and proteomic data indicates a partial gene knockdown, implying that a non-zero flux could occur through the predicted reactions. The resulting yield space for indigoidine production is now different from what was predicted by MCS algorithm (Fig. 3d). The yield space for the 14 gene set or the minimal set of 9 genes verified by RNAseq/proteomics are both similarly unconstrained. This suggests that partial implementation of the MCS-based predictions was still successful, as we observed in a shift of production from stationary to exponential phase while maintaining an improved indigoidine TRY. It was formally possible that glutamine titers could have increased, but not been detected if conversion to indigoidine was rate limiting. While we did not encounter this potential false-negative, it is an important caveat to consider when growth coupling to a precursor is the only feasible strategy. The observed shift in indigoidine production from stationary to exponential phase is also consistent with growth coupling.

Even with the limitations described above, our approach also allowed us to achieve, in one cycle of strain engineering, a high and consistent TRY for indigoidine from glucose across cultivation scales. With improvements in genetic tools and metabolic models it may be possible to further approach 90% MTY as predicted by the MCS algorithm. A better understanding of the terminator sequence efficiency (as observed in this study and elsewhere in *E. coli*^33^) would enable more efficient CRISPR mediated gene knockdown. Similar fold repression of targeted proteins by CRISPRi/dCas9 was recently reported^38^, suggestive of a limitation for existing CRISPR systems in *P. putida*. The plasmid-based CRISPRi system retained stable phenotype for 6 days, but can be further stabilized using genomic integration of the dCpf1/CRISPRi system or by developing multiplex gene deletion strategies^54,55^. Directly targeting proteins for degradation in a multiplex format^56^ could eventually be applied to prokaryotes^57^ and would sidestep the reliance on variable protein turnover kinetics. Additional reduction of competing reactions that draw on glutamate might only have a negligible impact on predicted indigoidine titer (Supplementary Discussion 1). As mechanistic studies using these foundational strains enable more refined genome scale models and generate informative datasets, more accurate metabolic flux modeling and machine learning approaches^58^ could in turn generate higher fidelity predictions for metabolic rewiring.

Selecting the best host-final product pair is crucial to developing the ideal production platform, and provides a key consideration in broadening our approach to additional studies. In earlier reports (Supplementary Table 7), high indigoidine production was achieved in the oleaginous yeast *Rhodosporidium toruloides* but remained low in *S. cerevisiae*, despite similar optimization of cultivation parameters^59–61^. This empirical comparison highlights the innate metabolic potential of a given host, and is consistent with our host-constrained calculated maximum theoretical yields for indigoidine (Supplementary Table 2). For indigoidine, the MTY from glucose in *P. putida* is 0.54 mol/mol and is comparable to that for *R. toruloides* (0.5 mol/mol), while *E. coli* (0.4 mol/mol) and *S. cerevisiae* (0.079 mol/mol) are lower. Genome scale metabolic models are now available for over 500 bacterial and eukaryotic organisms^62^ and we can assess the extent to which such multi-gene engineering would be useful for a target across hosts.

We propose using our workflow to calculate the % MTY for any given host-product as an early decision point to quickly filter for viable host-product pairs. It is likely that a given target will not only have different MTY limits across different hosts, but that the constraints will be different across targets. To show that our strategy is implementable for other targets, we calculated the maximum achievable yields for other product pairing regimes, and include the complete gene set required to implement two additional targets: a biodiesel precursor methyl ketone^24^ and a platform amino acid, arginine^63^ (Supplementary Data 3). Our preliminary results show that while indigoidine could be mathematically coupled up to 90% MTY, for methyl ketones it would be limited to 80%, and for an essential amino acid like arginine it would be 50% MTY. However, a subset of metabolites cannot be growth coupled using the MCS algorithm^8^. For final products derived from this class of metabolites, alternate hosts could be explored. Approaches using tools from synthetic biology, altered enzymatic functions^64,65^ or alternate homologs for growth paired steps (i.e., redox balance and ATP production)^66^, may also overcome these limitations. We also do not take into consideration products or intermediates that may be toxic. Industrial processes use renewable carbon sources that may contain growth inhibitory byproducts^67^. To solve these issues, tolerance engineering^68^ or adaptive laboratory evolution (ALE)^69^ could be useful. Production paired growth also enables powerful strategies like ALE to be used for direct improvement of production. The indigodine production system described here is of immediate interest to the biotech industry, and our methodology provides an avenue for the rapid prototyping of other scalable microbial production systems.

## Methods

### Computation of constrained minimal cut sets

*Pseudomonas putida* KT2440 genome scale metabolic model (GSMM) iJN1462^16^ was used. Aerobic conditions with glucose as the sole carbon source were used to model growth parameters. The ATP maintenance demand and glucose uptake were 0.97 mmol ATP/gDW/h and 6.3 mmol glucose/gDW/h, respectively. Constrained minimal cut sets (cMCS) were calculated using the MCS algorithm^8^ available as part of CellNetAnalyzer (version 2018.2). Excretion of byproducts was initially set to zero, except for the reported overflow metabolites for secreted products specific to *P. putida* (gluconate, 2-ketogluconate, 3-oxoadipate, catechol, lactate, methanol, CO_2_, and acetate). Additional inputs including minimum demanded product yield (10, 50, 70, 80, and 85 of MTY) and minimum demanded biomass yield at 10 or 25% of maximum biomass yield were also specified in order to constrain the desired design space. The maximum size of MCS was kept at the default (50 metabolic reactions). Knockouts of export reactions and spontaneous reactions were not allowed. The algorithm computed all minimal combinations of reaction knockouts blocking all undesired flux distributions and maintaining at least one of the desired metabolic flux distributions. With the specifications used herein each calculated knockout strategy (cMCS) demands production of glutamine even when cells do not grow. All cMCS calculations were done using API functions of CellNetAnalyzer^70^ on MATLAB 2017b platform using CPLEX 12.8 as the MILP solver. A summary of 417 common metabolites with the respective number of cut sets and number of targeted reactions to satisfy coupling restraints is included (Supplementary Fig. 5). Once the cMCS were enumerated, we used the decision workflow (Fig. 1a) to identify an optimal engineering strategy, from 63 different cMCS computed for glutamine, for experimental validation (Supplementary Table 3). Refer to Supplementary Method 1 for OptKnock implementation.

### Constraint-based methods to confirm the cMCS

iJN1462 was extended to account for indigoidine biosynthesis pathway and checked for strong growth coupling to confirm the chosen engineering strategy for experimental implementation.

The cytosolic reaction added for indigoidine biosynthesis from glutamine was as follows:

1 $$\, 2\,{\mathrm{L}} - {\mathrm{glutamine}} + 2\,{\mathrm{ATP}} + 2\,{\mathrm{Coenzyme}}\,{\mathrm{A}} + 2\,{\mathrm{FMN}} + 2.5\,{\mathrm{O}}2\\ \quad\, - > 2\,{\mathrm{Adenosine}}\,3^{\prime} ,5^{\prime} - {\mathrm{bisphosphate}}\\ \quad\,+ 2\,{\mathrm{FMNH}}2 + 2\,{\mathrm{Diphosphate}} + 2\,{\mathrm{AMP}} + 2\,{\mathrm{Pantetheine}} + {\mathrm{H}}2{\mathrm{O}}\\ \quad\,+ 2\,{\mathrm{Phosphate}} + {\mathrm{Indigoidine}}$$

Flux balance analysis (FBA) was used to calculate the maximum theoretical yield (MTY) from reaction stoichiometry and redox balance and also for single-gene deletion analysis. Flux variability analysis (FVA) was used along with FBA to check for minimum and maximum glutamine or indigoidine flux under the identified cMCS strategy to confirm product obligatory growth. FVA was performed with maximization of biomass formation as the objective function and the proposed gene deletions in each cMCS strategy along with constraints that were used for cMCS calculations. A positive minimum and maximum flux through the exchange reaction for the metabolite of interest (glutamine or indigoidine) confirmed production obligatory growth. COBRA Toolbox v.3.0^71^ in MATLAB R2017b was used for FBA and FVA simulations with the GLPK (https://gnu.org/software/glpk) or Gurobi Optimizer 8.1 (http://www.gurobi.com/) as the linear optimization solver. Production envelope was obtained using the internal COBRA Toolbox function, productionEnvelope(), and plotted for *P. putida* (Fig. 3d) as a fraction of maximum theoretical product yield on *y*-axis and maximum theoretical biomass yield on *x*-axis. Custom code used in this study is available as Supplementary Data 4.

### Reagents and culture conditions

All chemicals and reagents were purchased from Sigma-Aldrich (St. Louis, MO) unless mentioned otherwise. When cells were cultivated in a microtiter plate format, plates were sealed with a gas-permeable film (Sigma-Aldrich, St. Louis, MO). Tryptone and yeast extract were purchased from BD Biosciences (Franklin Lakes, NJ). Engineered strains were grown on M9 Minimal Media^72^ as described (15 mM (NH_4_)_2_SO_4_, 47.9 mM Na_2_HPO_4_, 22 mM KH_2_PO_4_, 8.56 mM NaCl, 2 mM MgSO_4_, 100 µM CaCl_2_) with the following modifications. Carbon sources (glucose or galactose) were used at 56 mM unless indicated otherwise. Trace minerals were purchased from Teknova Inc (Hollister, CA) and used diluted 2000-fold.

### Strains and strain construction

*Pseudomonas putida* KT2440 was used as the host for strain engineering. All strains used in this study are described in Supplementary Table 8. Specific DNA sequences used to design the gRNA array are described in Supplementary Data 1. All primers used in this study are listed in Supplementary Table 9. Electroporation with the respective plasmid was performed using a Bio-Rad (Bio-Rad Laboratories, Hercules, CA) MicroPulser preprogrammed EC2 setting (0.2 cm cuvettes with 100 µL cells, ~5 ms pulse and 2.5 kV) with slight modifications^73^. Cells transformed with replicative plasmid DNA were allowed to recover at 25 °C for 2.5 h before plating on selective agar media at 23 ˚C for overnight incubation. Cells transformed with non-replicative (integrating) plasmids were allowed to recover for 4–8 h in LB media before plating on selective agar media at 23 ˚C for an additional 24 h. Kanamycin sulfate or gentamicin sulfate (Sigma-Aldrich, St. Louis, MO) was used at a concentration of 50 µg/mL or 30 µg/mL, respectively. Integration of the *Escherichia coli galETKM* operon or the heterologous indigoidine gene pathway was implemented using a kanamycin/sucrose-counterselection plasmid for allelic exchange^74^. After confirming sucrose resistance and kanamycin sensitivity by patching clones onto solid agar media, correct clones were confirmed for the genotype by colony PCR using Q5 Polymerase enzyme (NEB, Ipswitch, MA). The dCpf1/CRISPRi system was adapted for use in *P. putida* by subcloning an endonuclease dead *Francisella tularensis subsp. Novicida cpf1*^75^ into a pBBR1 backbone and placed under the LacUV5 promoter. The synthetic gRNA array was constructed using gene synthesis techniques (Genscript, Piscataway, NJ) and cloned into the dCpf1/CRISPRi backbone using isothermal DNA assembly. All plasmid constructs were verified with Sanger sequencing before transformation into *P. putida*.

### Analytics and quantification using HPLC

Glucose and organic acids from cell cultures were measured by an 1100 Series HPLC system equipped with a 1200 Series refractive index detector (RID) (Agilent Technologies, Santa Clara, CA) and Aminex HPX-87H ion-exclusion column (300 mm length, 7.8 mm internal diameter). 300 µL aliquots of cell cultures were removed at the specified time points during production and filtered through a spin-cartridge (PALL Corporation, Port Washington, NY) with a 0.45-μm nylon membrane, and 10 μL of the filtrate was eluted through the column at 50 °C with 4 mM sulfuric acid at a flow rate of 600 μL/min for 30 min. Metabolites were quantified with an external standard calibration with authentic standards.

### Indigoidine extraction and quantification

Indigoidine was purified from *P. putida* with slight modifications as previously described^76^. Briefly, indigoidine is insoluble in most aqueous solutions and organic solvents except for dimethyl sulfoxide (DMSO) and dimethylformamide. We purified indigoidine by solubilizing all other materials with sequential resuspensions using different solvents. Cells were lysed by vortexing cells in 1% SDS and 100 mM NaCl and then centrifuged at 14,000 x *g* for 3 min. The supernatant was discarded and the pellet was washed with three rounds of methanol, isopropanol, water, ethanol, and hexane to remove contaminating proteins and metabolites. The pellet was allowed to dry overnight and then resuspended in DMSO at a final concentration of 1 mg/mL. Indigoidine purity was characterized by nuclear magnetic resonance. The sample was protected from direct exposure to sunlight to avoid photo-bleaching. A standard curve correlating indigoidine concentration to OD_612_ was generated using this reagent (Supplementary Fig. 6) by generating a linear regression to fit the data using GraphPad Prism 8 (Graphpad Software, San Diego CA). The purity of extracted indigoidine (Supplementary Fig. 7) from both *E. coli* and *P. putida* were cross-validated by ^1^H-NMR^61^.

To rapidly quantify indigoidine production in a high-throughput manner, a colorimetric assay was used as a proxy for indigoidine titer. Briefly, 100 µL of cells were pelleted by centrifugation at 20,000 x *g* for 2 min. The supernatant was discarded and 500 µL DMSO was added to the pellet. The solution was vortexed vigorously for 10 min to dissolve indigoidine. After centrifugation at 20,000 x *g* for 2 min, 100 μL of DMSO extracted indigoidine was added to 96-well flat- bottomed microplates (BD Biosciences, San Jose CA). Indigoidine was quantified by measuring the optical density (OD_612_) using a microplate reader (Molecular Devices, San Jose, CA) preheated to 25 °C and applying a standard curve generated from indigoidine. The equation used was

2 $${\mathrm{Y}}\,\left( {{\mathrm{g}}/{\mathrm{L}}\,{\mathrm{of}}\,{\mathrm{Indigoidine}}} \right) = 0.212 \ast {\mathrm{OD}}_{612} - 0.0035,$$

which was derived by averaging the standard curves generated from both the *E. coli* and *P. putida* biosynthetic indigoidine samples. The DMSO-solubilized cell lysate from wild-type *P. putida* does not contribute detectable signal when measured at OD_612_.

To correlate indigoidine yields with biomass yields, the dry cell weight was determined using OD_600_ to biomass conversion estimates^77^. 1.0 OD_600_ was converted to 0.38 g/L of dry cell weight.

### RNAseq and data analysis

Total RNA was prepared by Trizol-based RNA extraction^78^. RNA from trizol treated lysates were bound to a silica column (Direct-zol RNA MiniPrep Plus Kit, Zymo Research, Irvine CA) and its integrity confirmed using a Bioanalyzer RNA 6000 Nano assay (Agilent Technologies, Santa Clara, CA). rRNA was removed from 100 ng of total RNA using Ribo-Zero(TM) rRNA Removal Kit (Illumina Biotechnology, San Diego, CA). Stranded cDNA libraries were generated using the Illumina Truseq Stranded mRNA Library Prep kit. The rRNA depleted RNA was fragmented and reversed transcribed using random hexamers and SSII (Invitrogen-ThermoFisher, Carlsbad, CA) followed by second strand synthesis. The fragmented cDNA was treated with end-pair, A-tailing, adapter ligation, and ten cycles of PCR amplification. Prepared libraries were quantified using KAPA Biosystem’s next-generation sequencing library qPCR kit (Kapa Biosystems/Roche AG, Basel, Switzerland) and run on a Roche LightCycler 480 real-time PCR instrument. Sequencing of the flowcell was performed on the Illumina NovaSeq sequencer using NovaSeq XP V1 reagent kits, following a 2x150nt indexed run protocol. Reported gene expression values are the total normalized transcripts per kilobase million (TPM). Volcano plots and statistical analysis was conducted using the Geneious Differential Expression package in Geneious Prime (www.geneious.com). *P*-values were calculated assuming a binomial distribution and a random sampling model using Fisher’s exact test was applied. All raw data is available through NCBI-SRA associated with NCBI-Bioproject or via JGI (refer to Data Availability below).

### Targeted proteomics by LC-MS/MS

A targeted SRM (selected reaction monitoring) method was used to quantify relative levels of pathway proteins in samples under the various tested conditions in a 60 mL cultivation format. At the time points indicated, 1 mL of each sample was pelleted by centrifugation at 20,000  x *g* and flash frozen with liquid nitrogen at − 80 °C until ready for processing. Cells were lysed in 100 mM NaHCO_3_ using 0.1 mm glass beads in a Biospec Beadbeater (Biospec Products, Bartlesville, OK) with 60 s bursts at maximum power and repeated three times. Cell lysates were cooled on ice between each round. The clarified supernatant was harvested by centrifugation at 20,000 x *g*. The lysate protein concentration was estimated following the manufacturer’s directions for the BCA method (ThermoFisher Scientific/Pierce Biotechnology, Waltham, MA). Proteins were quantified for analysis using a SRM-targeted proteomic assay^79,80^. The SRM methods and data are available at Panoramaweb [https://panoramaweb.org/genome-scale-rewiring-indigoidine.url].

### Cultivation at different laboratory scales

Cultures from glycerol stocks were struck to single colonies on LB agar media with the appropriate antibiotic as necessary. Single colonies were used to inoculate overnight cultures in LB with the appropriate antibiotic. Saturated overnight LB cultures were then back-diluted 1/100x into M9 minimal media with the appropriate carbon source as indicated. Cultures were back-diluted and adapted twice to ensure robust cell growth before heterologous pathway induction. Adaptation of *P. putida Ec.galETKM* strains for growth in M9 minimal salt media with galactose had a long initial adaptation phase of around 96–120 h before cultures showed turbidity. All cultures were incubated with shaking at 200 rpm and 30 °C. To prepare cells for pathway induction, M9 adapted cultures were back-diluted to a starting OD_600_ of 0.1, at which point IPTG and arabinose were added as appropriate. Production cultures grown in 24-well deep-well plates (Axygen Scientific, Union City, CA) inoculated into a 200 µL culture volume and incubated InFors Multitron HT Double Stack Incubator Shaker (Infors HT, Bottmingen-Basel, Switzerland) set to 999 rpm linear shaker, and 70% humidity. For shake flask experiments, 60 mL cultures were grown in 250 mL unbaffled Erlenmeyer shake flask and incubated at 200 rpm with orbital shaking. For all experiments, the indigoidine pathway was induced with 0.3% w/v l-arabinose, and dCpf1 mediated gene repression was induced with 500 µM IPTG. Production assays were performed in independent biological triplicate and repeated at least twice, except for the scale-up experiments (described below), which were performed in biological duplicate. Standard error or standard deviation from the mean are shown as indicated and were calculated using GraphPad Prism 8. Preparation of cellular growth curves are described in **(**Supplementary Method 2).

### Batch experiments at 2 L bioreactor scale

Batch experiments were performed using a 2 L bioreactor equipped with a Sartorius BIOSTAT B® fermentation controller (Sartorius Stedim Biotech GmbH, Goettingen, Germany), fitted with two Rushton impellers fixed at an agitation speed of 800 rpm. Initial reactor volume was 1 L M9 Minimal Media (10 g/L Glucose, 0.3% w/v l-arabinose, 30 mM NH_4_^+^), and 50 mL overnight pre-culture in the same media. Feeding solution contained 100 g/L glucose, 300 mM NH_4_^+^ along with l-arabinose and kanamycin. The temperature was held constant at 30 °C. The bioreactor pH was monitored using the Hamilton EasyFerm Plus PHI VP 225 Pt100 (Hamilton Company, Reno, NV) and was maintained at a pH of 7 using 10 M sodium hydroxide. Dissolved oxygen concentration was monitored using Hamilton VisiFerm DO ECS 225 H0.

### 250 mL ambr® 250 bioreactor cultivations

Fed-batch bioreactor experiments were carried out in a 12-way ambr® 250 bioreactor system equipped with 250 mL single-use, disposable bioreactors (microbial vessel type). The vessels were filled with 150 mL M9 minimal salt media containing 10 g/L glucose as carbon source. Temperature was maintained at 30 ˚C throughout the fermentation process and agitation was set constant to 1300 rpm. Airflow was set constant to 0.5 VVM based on the initial working volume and pH was maintained at 7.0 using 4 N NaOH. Reactors were inoculated manually with 5 mL of pre-culture cell suspension. After an initial batch phase of 12 h, cultures were fed with a concentrated glucose feed solution (600 g/L glucose, 120 g/L ammonium sulfate, 50 µg/mL kanamycin, 3 g/L arabinose and 500 µM IPTG) by administering feed boluses every 2 h restoring glucose concentrations to 10 g/L (feed parameters: 3.1 min @ 50 mL/h). After observing glucose accumulation, feed addition was paused and resumed at reduced feed rates when glucose levels dropped below 10 g/L (1 min @ 50 mL/h). Experiments with a continuous feeding regime were initially fed at 1.3 mL/h (0.3 mL/h after seeing glucose accumulation). Samples were taken 1–2 times every day (2 mL) and stored at −20 °C. The ambr® 250 runtime software and integrated liquid handler was used to execute all process steps unless stated otherwise.

### Reporting summary

Further information on research design is available in the Nature Research Reporting Summary linked to this article.

## Supplementary information

Supplementary Information

Peer Review File

Reporting Summary

Description of Additional Supplementary Files

Supplementary Data 1

Supplementary Data 2

Supplementary Data 3

Supplementary Data 4

## Data Availability

Data supporting the findings of this work are available within the paper and its Supplementary Information files. A reporting summary for this Article is available as a Supplementary Information file. Datasets and strains analyzed or generated during the current study are available from the corresponding author upon request. All RNAseq raw data is available through NCBI-SRA associated with NCBI-Bioproject accession PRJNA580539-PRNJA580574 or at the JGI Genome Portal through Project ID 505977 [https://genome.jgi.doe.gov/portal/AssofEfficiency/AssofEfficiency.info.html]. The SRM methods and proteomics data are available at Panoramaweb [https://panoramaweb.org/genome-scale-rewiring-indigoidine.url]. All strains used in this study are described in Supplementary Table 8. All strains and plasmid sequences are available at The Joint BioEnergy Institute’s public Inventory of Composable Elements (ICE) [https://public-registry.jbei.org/login] after creating an account. Source data are provided with this paper.

## Source data

Source Data

## Code availability

All custom code used in this study is available in Supplementary Data 4.
