# Supplementary material for: Genome-scale metabolic rewiring improves titers rates and yields of the non-native product indigoidine at scale
Source: Nat Commun. 2020 Oct 23;11:5385. doi: 10.1038/s41467-020-19171-4 (PMC7584609; doi:10.1038/s41467-020-19171-4)
Supplement: Supplementary file 1 — Supplementary Information [file 41467_2020_19171_MOESM1_ESM.pdf]

Genome-scale metabolic rewiring improves titers rates and yields of the non-native product indigoidine at scale

Banerjee and Eng *et al*.

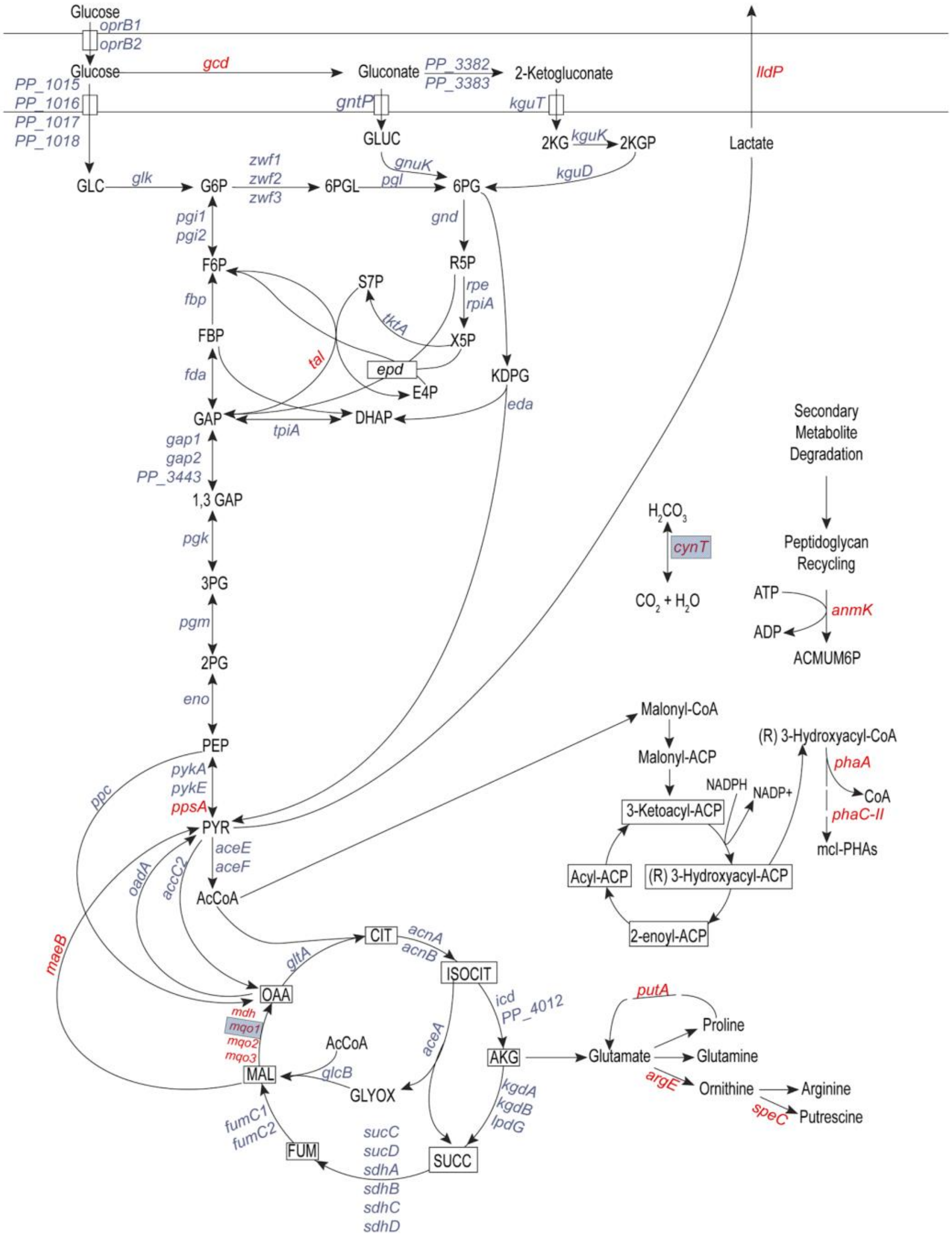

Supplementary Figure 1. Expanded metabolic map of cMCS prediction needed to generate growth coupled production of indigoidine from glucose. Related to Figure 1. A total of 14 genes were targeted for CRISPR interference excluding *mqo-I* and *cynT*. Targeted genes responsible for the requisite metabolic reactions indicated from the cMCS analysis are indicated in red. Genes excluded from targeted inhibition are further marked with blue boxes.

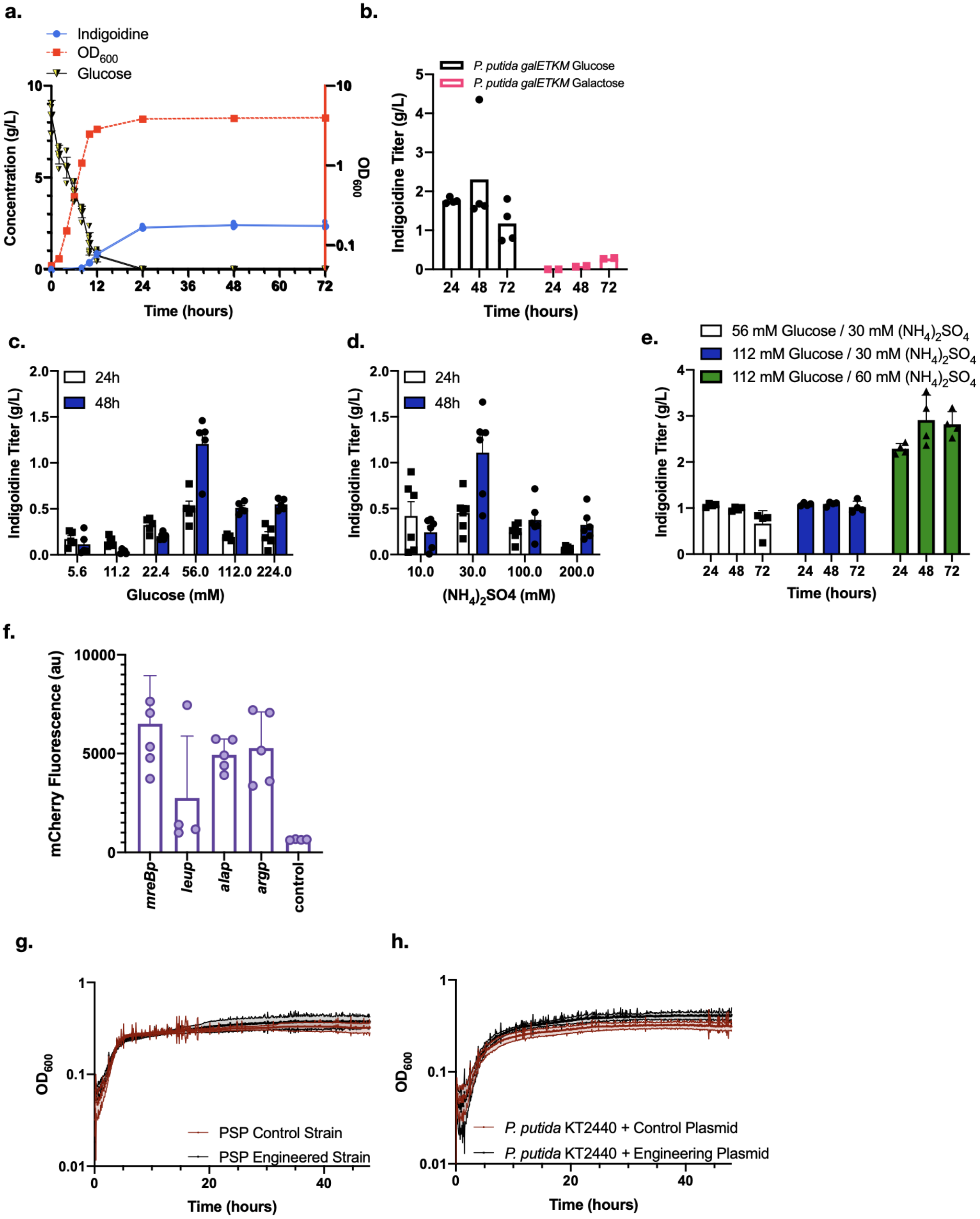
a.
Indigoidine
OD600
Glucose
Concentration (g/L)
OD600
Time (hours)
b.
P. putida galETKM Glucose
P. putida galETKM Galactose
Indigoidine Titer (g/L)
Time (hours)
c.
24h
48h
Indigoidine Titer (g/L)
5.6
11.2
22.4
56.0
112.0
224.0
Glucose (mM)
d.
24h
48h
Indigoidine Titer (g/L)
10.0
30.0
100.0
200.0
(NH4)2SO4 (mM)
e.
56 mM Glucose / 30 mM (NH4)2SO4
112 mM Glucose / 30 mM (NH4)2SO4
112 mM Glucose / 60 mM (NH4)2SO4
Indigoidine Titer (g/L)
Time (hours)
f.
mCherry Fluorescence (au)
mreBp
leup
alap
argp
control
g.
OD600
PSP Control Strain
PSP Engineered Strain
Time (hours)
h.
OD600
P. putida KT2440 + Control Plasmid
P. putida KT2440 + Engineering Plasmid
Time (hours)

Supplementary Figure 2. Characterization of indigoidine production kinetics in *Pseudomonas putida*.

(a) Kinetic time course of indigoidine production in *P. putida* KT2440. 60mL Cells were cultivated in M9 minimal media under standard conditions. Samples were harvested at the time points indicated to monitor optical density, extracellular glucose, and indigoidine. Sample time points are indicated as hours after 0.3% (w/v) arabinose induction.

(b) Comparison of indigoidine production in *P. putida galETKM* cultivated in 1% glucose or 1% galactose in M9 minimal salt media.

(c-e) Comparison of Carbon/Nitrogen (C/N) ratios for glucose and ammonium sulfate. *P. putida KT2440* carrying an genomically integrated heterologous indigoidine production pathway was cultivated in M9 minimal salt media where the glucose or ammonium sulfate concentration was varied as indicated. Samples were harvested 24 h or 48 h post induction with 0.3% (w/v) arabinose and indigoidine titer was measured as described in Methods. In (c), the same C/N ratio was compared but at two different concentrations of glucose and ammonium sulfate. Data are presented as mean ± SEM for n=5 biologically independent samples.

(f) Evaluation of native tRNA promoters. One hundred (100) bp promoter sequences from *P. putida* KT2440 tRNA ligases immediately upstream of the start ATG were amplified and cloned upstream of a *RBS-mCherry* gene cassette. Constructs were transformed into KT2440 grown in M9 minimal media with three rounds of adaptation. mCherry signal was determined 24 hours of growth in a 24 well deep well plate in a Spectramax m2e plate reader. mCherry fluorescence was compared against background fluorescence in a control strain harboring an empty vector control (pTE219). Data are presented as mean ± SD for n=5 biologically independent samples except for the *leu* promoter plasmid and empty vector control, which were n=4.

(g) Kinetic analysis of *P. putida* KT2440 indigoidine production strains for growth measured at OD_{600} with multiplex dCpf1/CRISPRi plasmids. After transforming the production strain with either the multiplex dCpf1/CRISPRi plasmid or a control dCpf1/CRISPRi plasmid, strains were adapted to growth in M9 1% glucose minimal salt media and then back diluted 1/100x into fresh growth media with 500 µM IPTG in a 96 well plate format. The OD_{600} was measured every 5 minutes for the duration of the 48 hour incubation period.

(h) Kinetic analysis of *P. putida* growth as in (g), but in wild-type *P. putida* (ie, lacking the indigoidine production pathway).

Source data are provided as a Source Data file.

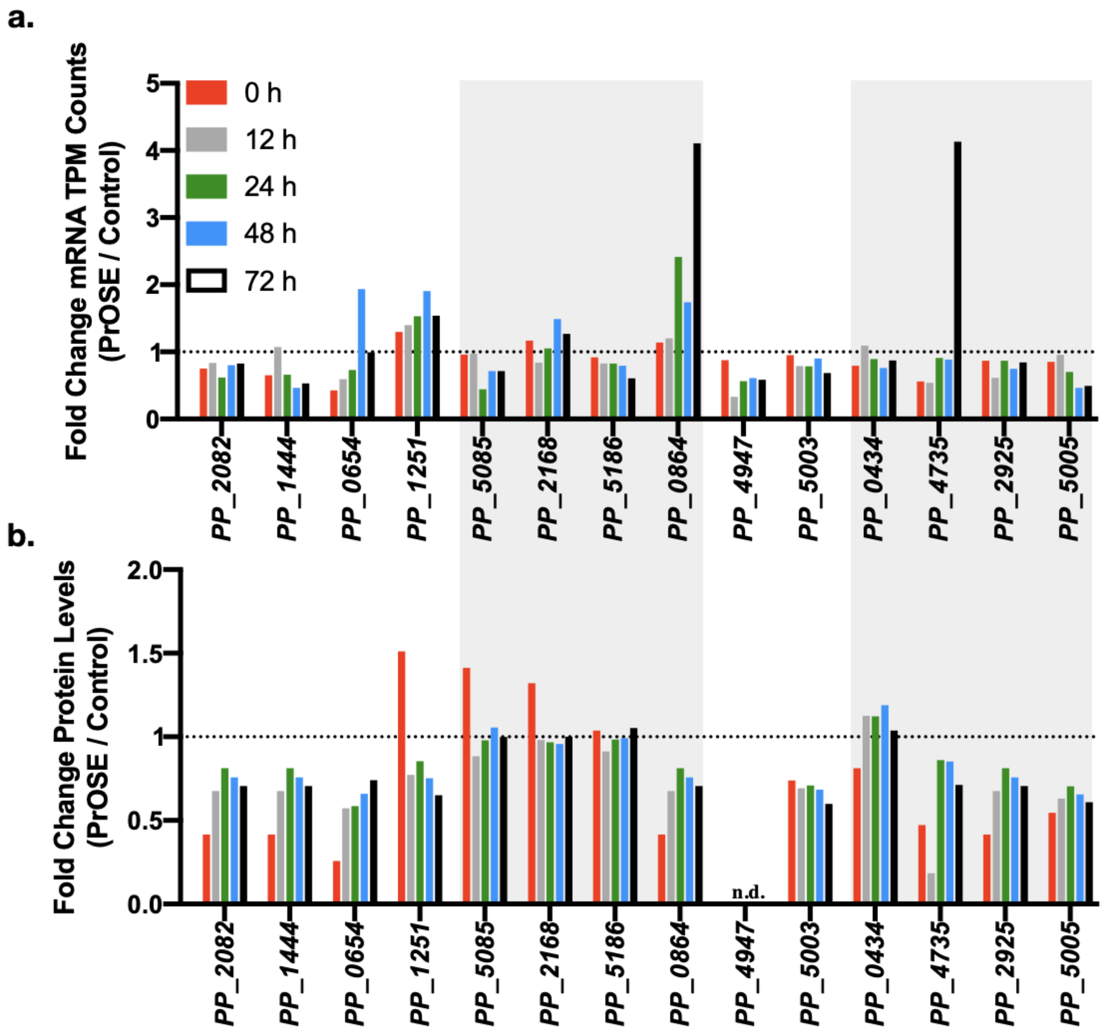

Supplementary Figure 3. Quantification of CRISPRi efficacy in *P. putida*. RNAseq (a) and Proteomic (b) validation of dCpf1 multiplex targeted gene knockdown. *P. putida* strains harboring a genomically integrated indigoidine pathway and a plasmid-borne dCpf1/CRISPRi engineered system were prepared for the production of indigoidine (see Methods) and sampled at the indicated time points. No peptides from PP_4947p were detected with the LC-MS/MS method (n.d.). For RNAseq, values were calculated by determining the ratio of Transcripts Per Kilobase Million (TPM) counts for the engineered strain divided by the TPM for the control strain. Independent biological replicates for either the engineered or control strain were averaged before determining the ratio (n=6 for engineered, n=2 for t=0 h control, n=1 for t=12,24,48,72 h timepoints). Samples were analyzed similarly for proteomic analysis (n=4 for both engineered and control strains). Source data are provided as a Source Data file.

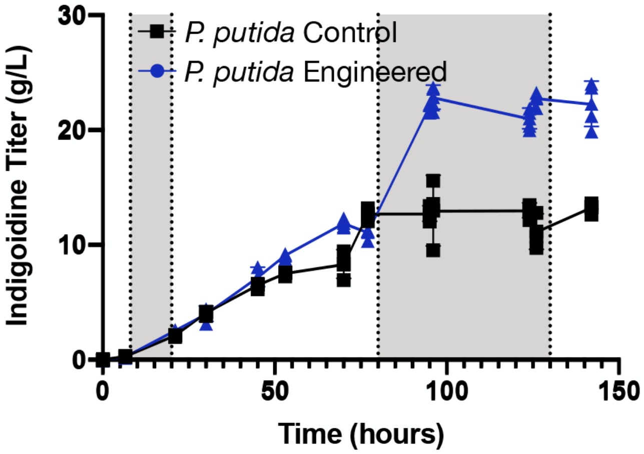

Supplementary Figure 4. Replicate fed-batch ambr250® cultivation of CRISPRi engineered product substrate paired indigoidine production strategy. Similar to the results described in Figure 3a, a replicate fed-batch feeding regime was implemented to demonstrate production of indigoidine during exponential phase growth. Instead of a bolus feeding regime as used in Figure 3a, this replicate tested a continuous feeding regime. A similar production of indigoidine during feeding was observed (second gray area on graph) when the control strain did not produce additional indigoidine. Data were analyzed using n>3 technical replicates of sampling from the same bioreactor and presented as mean ± SD. Source data are provided as a Source Data file.

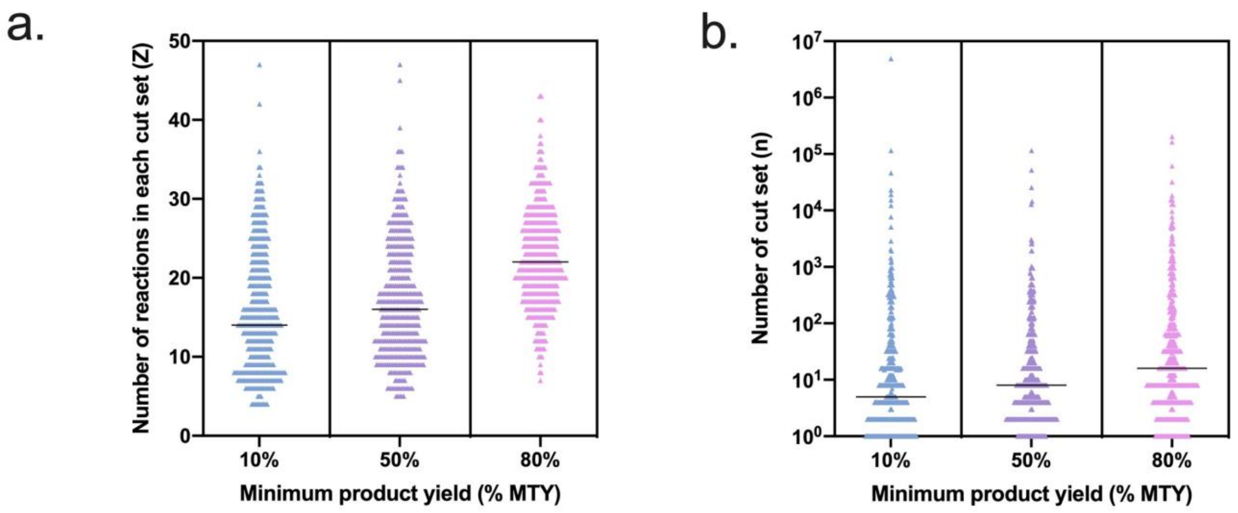

Supplementary Figure 5. Output from computational growth coupling metabolic modeling. cMCS predictions for 417 metabolites that could be coupled to at least 10% biomass yield and a minimum production yield of 10%, 50% and 80% MTY. The number of reactions in each cut set (Z) and number of cut sets (n) for each of the 417 metabolites is shown in a and b, respectively. Each metabolite is represented as a triangle and the number of cut sets is shown in blue whereas the size of the cut set is shown in pink. The black line marks the median for each case. Data was plotted using GraphPad Prism 8.

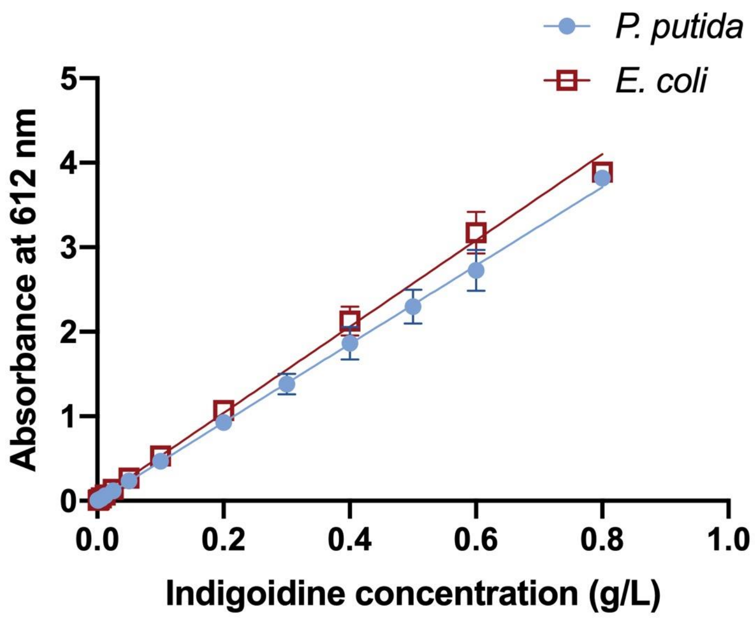

Strains	Equation	R^2
P. putida	Y = 0.212x - 0.0035	0.9911
E. coli	Y = 0.196x - 0.0035	0.9921

Supplementary Figure 6. Characterization of indigoidine. A standard curve used for indigoidine quantification relating absorbance at a wavelength of 612 nm to indigoidine concentration dissolved in DMSO. In each equation Y is indigoidine concentration in g/L and x is absorbance at 612 nm (see Methods). The plotted standard curve for indigoidine purified from *P. putida* was repeated >3 times over from freshly generated microbial cultures over the course of several months. A standard curve was also generated using the same pathway genes expressed in *E coli*. Data are presented as mean ± SD for n=6 independent absorbance measurements at each concentration. Raw values are included in the supplementary source file. R^2 value was calculated using GraphPad Prism 8. Source data are provided as a Source Data file.

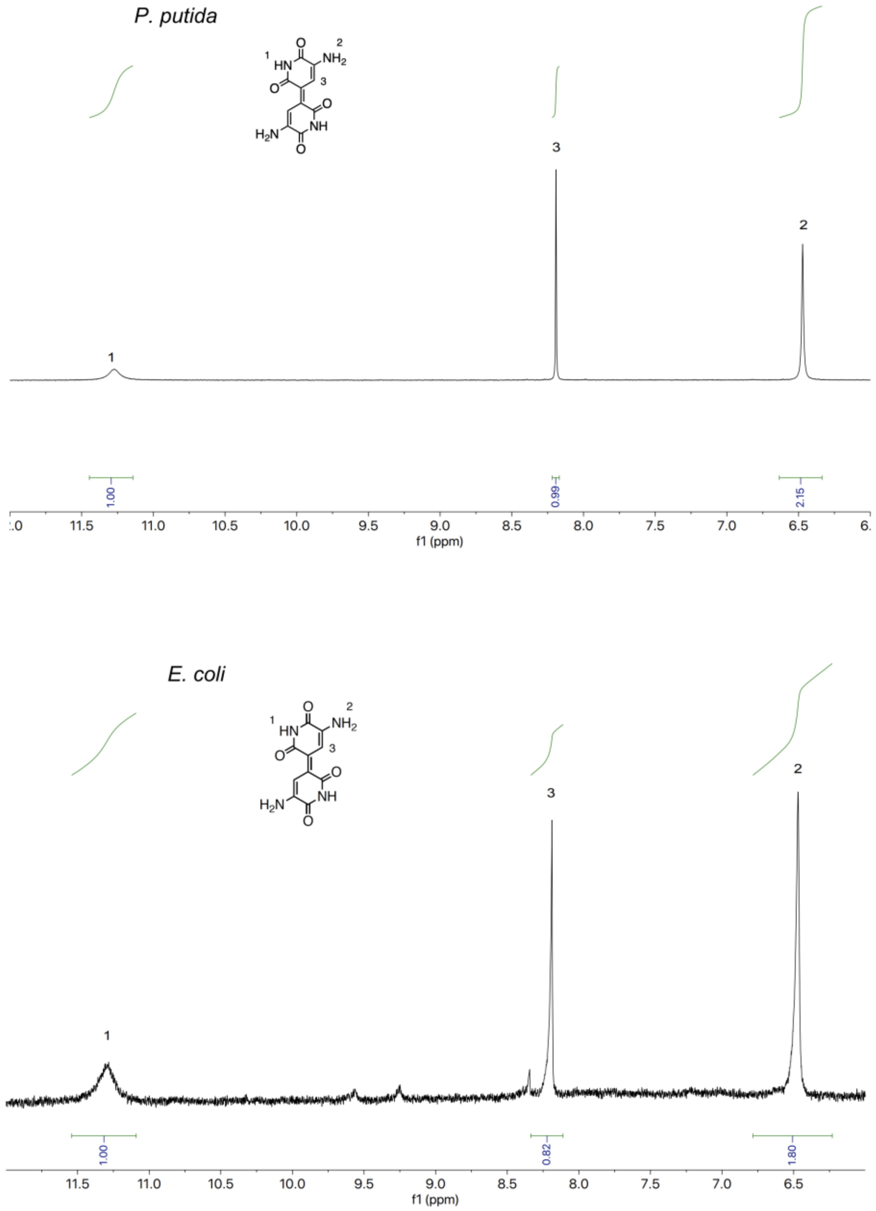

Supplementary Figure 7. Analysis of indigoidine purity by H-NMR. Indigoidine was extracted from *P. putida* and *E. coli* harboring expression the heterologous pathway and purified as described (see Methods). Indigoidine purified from either host shows similar purity. A representative trace from n=3 biologically independent samples is shown.

Supplementary Table 1. Potential for product substrate pairing for all metabolites in *P. putida* KT2440 and *E. coli* MG1655 using glucose as the sole carbon source.

Organism	*E. coli* MG1655[1]	*P. putida* KT2440	Remarks
Genome-scale metabolic model (GSMM)	iJO1366	iJN1462	
Glucose uptake limit (mmol/gDW/h)	15	6.3	
ATP maintenance (mmol/gDW/h)	3.15	0.92	
Total Reactions	2582	2928	
Repressible reactions	1414	2046	
Irrepressible reactions	1168	882	
Internal metabolites	1805	2145	892 metabolites present in *P. putida*, but absent in *E. coli*
Glucose-producible organic metabolites	954	979	
Minimum Metabolite yield (%)	10	10	
Metabolites with feasibility of strong coupling	954	966	98.6% in *P. putida* versus 99.4% in *E. coli*[1]
Minimum cMCS size	3	4	Metabolites including 4-Hydroxy-L-threonine, Mannose-6-phosphate, Mannuronate in *P. putida*
Maximum cMCS size	50	48	Metabolites including Vaccenyl coenzyme A in *P. putida*
Mean cMCS size	17.4	18.9	

Supplementary Table 2. Comparison of industrially relevant hosts for glutamine and indigoidine production with respect to maximum theoretical yields.

	P. putida	*C. glutamicum*	*E.coli*	*R. toruloides*	*S. cerevisiae*
**Genome-scale metabolic model (GSMM)**	iJN1462[2]	iCW773[3]	iML1515[4]	iRhto1108C[5]	iMM904[6]
**Glutamine MTY[a] (mol/mol glucose)**	1.141	1	1.135	1.118	0.481
**Biomass Yield (gDW/mmol glucose)**	0.098	0.092	0.088	0.075	0.029
**Indigoidine MTY[a] (mol/mol glucose)**	0.537	0.4[c]	0.4	0.503[c]	0.079[c]
**Glucose uptake rate[b] (mmol/gDCW/hr)**	6.3	4.67	10	5	10
**ATP maintenance demand[b] (mmol/gDCW/hr)**	0.92	0	6.86	1.012	1

[a]Maximum Theoretical Yield (MTY) calculated using [b]constraints for each Genome-scale Metabolic Model (GSMM) to best represent cell phenotype.
[c]No indigoidine formation if flavin mononucleotide (FMN) is limiting.

Supplementary Table 3. Analysis of all the computed cMCS in this study for growth coupled glutamine production as a proxy for indigoidine.

cMCS run	% MTY	% YBS	Number of cut sets (n)	Size of reaction cut set (Z)	Number of genes in cut set	Size of cut set <25	Small gene set	Gene KO congruent with rxn KO	Coupled indigoidine production	Number of essential genes according to RbTnSeq data	High product yield (% MTY)
1	10	10	8	24	28	Y	N	Y	Y	6	N
2	10	10	16	21	29	Y	N	N	NA	NA	NA
3	10	10	3	12	15	Y	Y	Y	Y	3	N
4	50	10	2	23	28	Y	N	N	NA	NA	NA
5	70	25	16	26	NA	N	N	N	NA	NA	NA
6	70	25	1	29	NA	N	N	N	NA	NA	NA
7	80	10	1	15	16	Y	Y	Y	Y	1	Y
8	80	10	2	23	31	Y	N	N	NA	NA	NA
9	85	10	12	17	17	Y	Y	N	NA	NA	NA
10	85	10	2	21	24	Y	N	N	NA	NA	NA
**Grand total cut sets**			**63**			**46**	**16**	**12**	**12**		**1**

[a]MTY: Maximum theoretical yield (mole of product per mole of glucose)
[b]YBS: Biomass yield (gDW per mmol of glucose)
[c]KO: Knockout

Supplementary Table 4. Analysis of suitable starting carbon sources to determine compatible carbon sources for cultivation for substrate-product pairing with indigoidine.

**Glucose/Indigoidine PrOSE modeling: acceptable alternate carbon sources**	**Glucose/Indigoidine PrOSE modeling: unacceptable alternate carbon sources**
Glycerol, Fructose, Mannose, Serine, Threonine, Galactose, Asparagine, Aspartate, Glycine, Homoserine, Xanthine	Lysine, Leucine, Succinate, Malate, Tryptophan, Tyrosine, Valine, Xylose, Proline, *p*-coumarate, Cysteine, α-ketoglutarate

Supplementary Table 5. Comparison of growth coupling strategies for α-ketoglutarate using OptKnock and cMCS algorithms to identify gene cut sets to improve indigoidine yield.

Algorithm	OptKnock[11]	cMCS[1]
Number of reactions for knockout	4	21
Reactions	GAPD/PGK, SUCDi, TALA/TKT1, TPI	ABTA, AGM3PA, AGM3PH, AGM4PA, AGM4PH, AKGDH, ALDD2x, DHORD2, FDH, GAD2ktpp, GLCNtex, LDH_D, LSERDHr, PDH, PDHcr, RBK, SUCD4, SUCDi, TALA, GLCabcpp/HEX1, MCITD/MCITL2/MCITS/MICITDr
YPS[a]	0.056 to 0.473	1.085
YBS[b]	0.012	0.0138

[a]YPS: Product yield (mole of product per mole of glucose)
[b]YBS: Biomass yield (gDW per mmol of glucose)

Supplementary Table 6. Comparison of growth coupling strategies for glutamine using OptKnock and cMCS algorithms to identify gene cut sets to improve indigoidine yield.

Algorithm	OptKnock[11]	cMCS[1]
Number of reactions for knockout	15	15
rxns	ACOAD3f, ALLTN, ECOAH20, ECOAH4, FMNRx2, GLYCLTDy, ME2, NADH16pp, OAADC, PGI, PIt2rpp, PPA,PPCK, PPKr, TRPS3	ANHMK, D_LACt2pp, GLCDpp, H2CO3D, HCO3E, MDH, MDH2, ME2, NACODA, ORNDC ,PHAPC40, PPS, PROD2 ,TALA
YPS[a]	0 to 0.76	0.92
YBS[b]	0.0105	0.0149
Indigoidine molar yields	<0.001	0.48

[a]YPS: Product yield (mole of product per mole of glucose)
[b]YBS: Biomass yield (gDW per mmol of glucose)

Supplementary Table 7. Comparison of industrially relevant hosts with respect to the highest experimentally reported indigoidine titers.

	P. putida	*C. glutamicum*[c]	*E. coli*	*R. toruloides*	*S. cerevisiae*
**Highest indigoidine titers (g/L)**	25.6 from glucose minimal media (this study)	n.d.	7.08 from rich media[7]	18.04 from rich complex media[8,9]	0.98 from rich complex media[10]
**Rate (g/L/h)**	0.22[a]	n.d.		0.15	
**Yields (g/g)**	0.33[b]	n.d.		0.19[8,9]	

[a] When cells are fed glucose from a continuous feeding regime (i.e. exponential growth phase).

[b] When cells are fed glucose in batch mode culture. (i.e. stationary phase).

[c] Refer to Supplementary Table 2 for theoretical yield calculations.

Supplementary Table 8. Strains used in this study.

**Strain number**	**Genotype**	**Relevant figure(s)**	**Reference**
JBEI-13809	*Pseudomonas putida* KT2440 wild type prototroph CmR AmpR	Supplementary Figure 1	Nieto *et al.*[12]
JBEI-137184[a] (TEAM-1120)	KT2440 *Pp_5402::arap-Sc.bpsA,Bc.sfp*	Supplementary Figure 1	This study
JBEI-137183[a] (TEAM-1411)	KT2440 *Pp_5402::arap-Sc.bpsA,Bc.sfp Pp_0871::galp-Ec.galETKM*	Supplementary Figure 1	This study
JBEI-137178 (TEAM-1361)	KT2440 {p/pTE328 *neo*[d] *BBR1 PP_0933(mreB)p-RBS-mCherry*}	Supplementary Figure 1	This study
JBEI-137179 (TEAM-1362)	KT2440 {p/pTE329 *neo BBR1 PP_4794(leu)p-RBS-mCherry*}	Supplementary Figure 1	This study
JBEI-137180 (TEAM-1363)	KT2440 {p/pTE330 *neo BBR1 PP_4474(ala)p-RBS-mCherry*}	Supplementary Figure 1	This study
JBEI-137181 (TEAM-1364)	KT2440 {p/pTE330 *neo BBR1 PP_5089(arg)p-RBS-mCherry*}	Supplementary Figure 1	This study
JBEI-136298[b]	KT2440 *Pp_5402::arap-Sc.bpsA,Bc.sfp* {p/pTE219 *neo BBR1 Placuv5-dCpf1 j23101p-nt_gRNA*}	Figure 2, Figure 3, Supplementary Figure 2	This study
JBEI-105555[c]	KT2440 *Pp_5402::arap-Sc.bpsA,Bc.sfp* {p/pTE327 *neo BBR1 lacuv5p-dCpf1* 14 gene CRISPRi array}	Figure 2, Figure 3, Supplementary Figure 2	This study
JBEI-136186[c] (TEAM-1425)	KT2440 *Pp_5402::arap-Sc.bpsA,Bc.sfp Pp_0871::galp-Ec.galETKM* {p/pTE219 *neo BBR1 Placuv5-dCpf1 j23101p-nt_gRNA*}	Figure 2, Figure 3	This study
JBEI-136187[b] (TEAM-1423)	KT2440 *Pp_5402::arap-Sc.bpsA,Bc.sfp Pp_0871::galp-Ec.galETKM* {p/pTE327 *neo BBR1 lacuv5p-dCpf1* 14 gene CRISPRi array}	Figure 2, Figure 3	This study
JBEI-18378	E. coli BL21(DE3) Δ*prp*RBCD::t7p-*sfp*,t7p-*prpE*	Supplementary Figure 4	Pfeifer *et al.*[13]

[a] Genomic integrations are targeted to an intergenic region adjacent to the indicated locus.
[b] nt: non-targeting gRNA sequence with no homology to the *P. putida* KT2440 genome.
[c] Refer to Supplementary Data 1 for sequences of targeted genes with sequences for targeting gRNAs, promoters, and terminators used in the multiplex array.
[d] *neo* is also known as APH(3')-II family aminoglycoside O-phosphotransferase and confers resistance to 50 µg/mL kanamycin in *P. putida*.

Supplementary Table 9. Primers used in this study.

Primer number	Sequence (5' - 3')	Usage notes	Related to strain
TEAM-673	CAACAGTAGAAATTCGGATCCATTATACCTAGG	FnCpf1 sequencing primer	JBEI-136298
TEAM-672	CGGGAAGACAGGAAGACTGCTGGAT	FnCpf1 sequencing primer	JBEI-136298
TEAM-671	GCTACAACCTGGATAAGGGCTACTTCG	FnCpf1 sequencing primer	JBEI-136298
TEAM-670	GGTCCACTTGCCCTTGGCTGCC	FnCpf1 sequencing primer	JBEI-136298
TEAM-669	CCGGCGCCAACAAGTTCAACGATG	FnCpf1 sequencing primer	JBEI-136298
TEAM-668	GCCTTCTCCTTCAGGAGCAGGTTGA	FnCpf1 sequencing primer	JBEI-136298
TEAM-667	GGCGCTAACAAGATGCTCCCAAAGG	FnCpf1 sequencing primer	JBEI-136298
TEAM-666	CGGATGGGTTGTAGAACTTGATGGACTTTG	FnCpf1 sequencing primer	JBEI-136298
TEAM-665	GATCGCCCCAAAGAACCTCGATAACCC	FnCpf1 sequencing primer	JBEI-136298
TEAM-664	CGGTCTTCTTGGCGATCAGCTCCT	FnCpf1 sequencing primer	JBEI-136298
TEAM-663	CAAGGGCTTCCACGAAAACCGC	FnCpf1 sequencing primer	JBEI-136298
TEAM-662	CGATGCGGTAGATGATAGAGGTTGGGATATC	FnCpf1 sequencing primer	JBEI-136298
TEAM-661	CATCGCCTTCTATCGCCTTCTTGACGAG	FnCpf1 sequencing primer	JBEI-136298
TEAM-660	CTCGTCAAGAAGGCGATAGAAGGCGATG	FnCpf1 sequencing Primer	JBEI-136298
TEAM-1774	agactatcggctgtagcctaggtatCCCAAAGGTTGGCAACCTGCTCCCGGCAACGGATAATGCCCAGTTTTTCCACCTGCGCATCCAGGCGCTCACCGCGCTTGCGGTCGAAAAGAGATCCCCGCAGAAGATTCAAAAT	arginine tRNA promoter gblock fragment	JBEI-137181

	GGTGAGGTGG		
TEAM-1773	agactatcggctgtagcctaggtatACACTGCATCGAGACTGCAGGACCGATATAATGCGGCCCTTTGCCGTCGTTTCGTCGACGCGTTTGCCCAACAGGGCCGCCGTTTCCGTGGAAGAACCTGCAGAAGATTCAAAATGGTGAGGTGG	alanine tRNA promoter gblock fragment	JBEI-137180
TEAM-1772	agactatcggctgtagcctaggtatGTATGCTACGGCGTTCACTCTAAACGACACCGGAGTGCCGGACGCAGCGAGGTTCCGCTGCCTGGAACAAGGTCTCTTCTCTCTCAGCCAAAAGTAGCCGCAGAAGATTCAAAATGGTGAGGTGG	leucine tRNA promoter gblock fragment	JBEI-137179
TEAM-1771	agactatcggctgtagcctaggtatCGGAATTTGGGTCCGCGGTGAGGAACATGTCAGCTTTCTGGCGGTCTAACGGCCGCGAAATTCTGCTGGCGTGCTCGGAAAAACAGCCAATTTAACATAGCAGAAGATTCAAAATGGTGAGGTGG	mreB promoter gblock fragment	JBEI-137178
TEAM-1770	AAAACAGCCAATTTAACATAGCAGAAGATTCAAAATGGTGAGGTGG	Gibson assembly primer for pTE328, backbone	Plasmid Backbone, JBEI-137178 - JBEI-137181
TEAM-1769	CACCGCGGACCCAAATTCCGatacctaggctacagccgatagt	Gibson assembly primer for pTE328, backbone	Plasmid Backbone, JBEI-137178 - JBEI-137181
TEAM-1701	GTACACCGCGGAGGCTCGATC	colony PCR primer for bpsA spf pTE302	JBEI-137184
TEAM-1700	tgctatggcatagcaaagtgtgacgc	colony PCR primer for bpsA spf pTE302	JBEI-137184
TEAM-1699	agggatgcacggcttcccgta	colony PCR primer for bpsA spf pTE302	JBEI-137184
TEAM-1698	cagttggacaaatccgatatccgctt	colony PCR primer for bpsA spf pTE302	JBEI-137184
TEAM-1697	gttctgcaacacgttgagccagc	colony PCR primer for bpsA spf pTE302	JBEI-137184
TEAM-1696	aatggctgtatgcgccgcacac	colony PCR primer for bpsA spf pTE302	JBEI-137184

TEAM-1695	aaccagacgctgttgagccagg	colony PCR primer for bpsA spf pTE302	JBEI-137184
TEAM-1694	gagcccagaatatccggaagatcgt	colony PCR primer for bpsA spf pTE302	JBEI-137184
TEAM-1693	gtgattaccttgatccatcagtgcagc	colony PCR primer for bpsA spf pTE302	JBEI-137184
TEAM-1692	gcttgtatcgaactgagcgaaaagga	colony PCR primer for bpsA spf pTE302	JBEI-137184
TEAM-1691	caccgttcgcacccagaactgc	colony PCR primer for bpsA spf pTE302	JBEI-137184
TEAM-1690	ggcaatccgctgccagcaagc	colony PCR primer for bpsA spf pTE302	JBEI-137184
TEAM-1689	cttcacgttccagacgacgtgcc	colony PCR primer for bpsA spf pTE302	JBEI-137184
TEAM-1688	gtgctggaaagcccgaccatcgaa	colony PCR primer for bpsA spf pTE302	JBEI-137184
TEAM-1687	accaatatcttcacgcagcaggc	colony PCR primer for bpsA spf pTE302	JBEI-137184
TEAM-1686	ctggtgaaacacatccgttatctgc	colony PCR primer for bpsA spf pTE302	JBEI-137184
TEAM-1685	CTAAAAAGCCGCCCTCTCAAGGC	colony PCR primer for bpsA spf pTE302	JBEI-137184
TEAM-1684	TTGCCAACAGGCCGAACAGTCAC	colony PCR primer for bpsA spf pTE302	JBEI-137184
TEAM-1674	caggtcgactctagaggatcCGAAGCGATAGCGCCCA	Construction of pTE302, 3' homology to PP_5402	JBEI-137184
TEAM-1673	gggcctttctgcgtttatacCGATACGAATCCGAGGCAAAAA	Construction of pTE302, 3' homology to PP_5402	JBEI-137184
TEAM-1672	TTTGCCTCGGATTCGTATCGgtataaacgcagaaaggcccac	Construction of pTE302, araC/sfp1/bpsA fragment from pTE252	JBEI-137184
TEAM-1671	CACTGGCAGCACGACGGTACcgtcttatgacaacttgacggctacatcattcact	Construction of pTE302,	JBEI-137184

		araC/sfp1/bpsA fragment from pTE252	
TEAM-1670	cgtcaagttgtcataagacgGTACCGTCGTGCTG	Construction of pTE302, 5' homology to PP_5402	JBEI-137184
TEAM-1669	attcgagctcggtacccgggAGAGTTGTCGCCAGATCAACT	Construction of pTE302, 5' homology to PP_5402	JBEI-137184
TEAM-1668	GTTGATCTGGCGACAACTCTcccgggtaccgagctcg	Construction of pTE302, backbone fragment (pK18mobsacB)	JBEI-137184
TEAM-1667	AACTGGGCGCTATCGCTTCGgatcctctagagtcgacctgc	Construction of pTE302, backbone fragment (pK18mobsacB)	JBEI-137184
TEAM-1603	GCGGACGCTGCCATCGGAAAG	colony pcr primers, galETKM	JBEI-137183
TEAM-1602	GCGACTTTACTTTCCGCCCGTAT	colony pcr primers, galETKM	JBEI-137183
TEAM-1601	ACGTTTGAAGTTACTGTTGATGATGACGAC	colony pcr primers, galETKM	JBEI-137183
TEAM-1600	GCTGATCGATTGCCGCTCACTGG	colony pcr primers, galETKM	JBEI-137183
TEAM-1599	CGGAGCGCAGCAGAGGCGGAT	colony pcr primers, galETKM	JBEI-137183
TEAM-1598	GGCGAAGAGAATCAACACTGGC	colony pcr primers, galETKM	JBEI-137183
TEAM-1597	CGGTGAAACCAGAATCCATTGCCC	colony pcr primers, galETKM	JBEI-137183
TEAM-1596	CGCCGCTACAACCCGCTCACCGGG	colony pcr primers, galETKM	JBEI-137183
TEAM-1595	CCGTAGCGTTTAGCCACAGGTGG	colony pcr primers, galETKM	JBEI-137183
TEAM-1594	GGCAACCAAGAAGTgtgCTCGCC	colony pcr primers, galETKM	JBEI-137183

TEAM-1593	GTGTGCCGCCGAGGAAGTTGC	colony pcr primers, galETKM	JBEI-137183
TEAM-1592	GAAGGTCTACACCACCGCTCCGGC	colony pcr primers, galETKM	JBEI-137183
TEAM-1591	CGTATGGAATTTTGGGCTGATCGCC	colony pcr primers, galETKM	JBEI-137183
TEAM-1590	GATTAGCGCCATGCGCGCCGCTA	colony pcr primers, galETKM	JBEI-137183
TEAM-1589	CGACCAGTACGATTCGTTCGTCG	colony pcr primers, galETKM	JBEI-137183
TEAM-1588	CGTGGCAAGCGCCCTGCACCAG	colony pcr primers, galETKM	JBEI-137183
TEAM-1587	attggtcgAGGCGGCCCCCAG	galETKM gibson assembly primers for integration at PP_0871	JBEI-137183
TEAM-1586	ATGATTTGAGACATCAAGTCGCTGTTTTTCTCG	galETKM gibson assembly primers for integration at PP_0871	JBEI-137183
TEAM-1585	CGACTTGATGTCTCAAATCATCTTTTAATGATAATAATTCTCATTATATTGCCGC	galETKM gibson assembly primers for integration at PP_0871	JBEI-137183
TEAM-1584	CTGCAGCATTGTCCGCGCACTGGT	galETKM gibson assembly primers for integration at PP_0871	JBEI-137183
TEAM-1583	CGGACAATGCTGCAGGCGTTGAAATAACT	galETKM gibson assembly primers for integration at PP_0871	JBEI-137183
TEAM-1582	CGGCGGCCGGACGCGGCGCC	galETKM gibson assembly primers for integration at PP_0871	JBEI-137183
TEAM-1581	CGCGTCCGGCCGCCGAAGCCCTGAAGca	galETKM gibson assembly primers for integration at PP_0871	JBEI-137183
TEAM-1580	GCCGCCTcgaccaattgctcaaagcctct	galETKM gibson assembly primers for integration at PP_0871	JBEI-137183

Supplementary Method 1. Implementation of Optknock.

OptKnock[11] was implemented on iJN1462 using COBRA Toolbox 3.0[14] on MATLAB 2017b platform. Gurobi Optimizer 8.1 (http://www.gurobi.com/) was used as the MILP solver. Additional constraints including aerobic condition, glucose uptake, ATP maintenance demand, excretion of byproducts were kept the same as used for cMCS computation (Main methods section). Export reactions and spontaneous reactions were excluded from the targetable reaction set. Additional constraints including maximum product yield (80% of MTY) and minimum demanded biomass yield at 10% of maximum biomass yield were also specified in order to constrain the desired design space. The maximum number of reaction deletions that were tested ranged from 3 to 20. Simulations were allowed to run upto 2 hrs, the same time assigned for cMCS computation. 40 solution sets with 11 to 18 deletions were found, all with ‘non unique’, not growth coupled, production phenotypes. None of them improved indigoidine production. The comparable solution set, although non-unique and not growth coupled, has been tabulated in **Supplementary Tables 5 and 6**.

Supplementary Method 2. Determination of cell growth or fluorescence with microtiter plate reader based assays.

Growth was monitored in a Molecular Devices (San Jose, CA) Spectramax m2e plate reader plate reader by measuring optical density at OD_{600} at 5-min intervals over a 48 hour interval. Strains of the appropriate genotype were struck to single colonies from glycerol stocks and first grown on LB agar media supplemented with the appropriate antibiotic and prepared for adaptation in liquid M9 media with 1% glucose as described for indigoidine production assays. The plate was sealed using a Breath-easy sealing membrane (Sigma-Aldrich) to ensure optimal oxygen exchange. Using the Softmax Pro plate v7.0 reader control package, the plate was incubated with constant shaking and the temperature was held to 30 ˚C for the duration of the experiment. For fluorescence quantitation of native P. putida promoters using detection of *RBS-mCherry*, cells of the appropriate genotype were prepared as above, but instead were cultivated in a 30˚C warm room using the same 24 well deep well plate used for indigoidine production assays. When cells had completed adaptation, cultures were back-diluted to an initial OD_{600} of ~0.1 and allowed to grow for 24 hours. 100 µL of each culture was then transferred to a 96 well microtiter dish and fluorescence using the monochromator was measured with a wavelength pair of 587 nm / 610 nm for excitation / emission.

Supplementary Discussion 1. Glutamate metabolism in *P. putida.*

In *P. putida* central metabolism, glutamate can be converted into α-ketoglutarate, fumarate, aspartate, proline, ornithine, and glutamine. These characterized pathways result in flux redistribution towards α-ketoglutarate, fumarate or aspartate, in addition to the pathways that are targeted as shown in **Supplementary Figure 1**. The first two metabolites (α-ketoglutarate, fumarate) redirect flux toward TCA whereas biosynthesis of aspartate would require equimolar amounts of oxaloacetate. If the biomass yields are the same as that of WT, based on biomass equation formulation used in the genome scale model, in the engineered strain about 3.3% glutamine and 4% (glutamate equivalents) accumulated intracellularly would be sufficient for 10% biomass yield. Using the experimentally observed indigoidine yields in WT and engineered *P. putida* strains, we find that only about 10% of MTY glutamine in WT is converted to indigoidine whereas 70% of the MTY glutamine was successfully converted to indigoidine in the engineered strain. The set of exchange reactions that might *in silico* impact growth coupled indigoidine production in the engineered strain are for pyruvate, α-ketoglutarate, leucine, valine, citrate, isocitrate and D-alanine. Biosynthesis of all these metabolites are upstream of glutamate. Our HPLC method did not detect any organic acids. Of the remaining three amino acids, leucine, valine and D-alanine are synthesized from pyruvate. Blocking these pathways above would generate auxotrophies for the indicated metabolites.

Supplementary References

1. von Kamp, A. & Klamt, S. Growth-coupled overproduction is feasible for almost all metabolites in five major production organisms. *Nat. Commun.* **8,** 15956 (2017).
2. Nogales, J. *et al.* High-quality genome-scale metabolic modelling of *Pseudomonas putida* highlights its broad metabolic capabilities. *Environ. Microbiol.* **22,** 255–269 (2020).
3. Zhang, Y. *et al.* A new genome-scale metabolic model of *Corynebacterium glutamicum* and its application. *Biotechnol. Biofuels* **10,** 169 (2017).
4. Monk, J. M. *et al.* iML1515, a knowledgebase that computes *Escherichia coli* traits. *Nat. Biotechnol.* **35,** 904–908 (2017).
5. Dinh, H. V. *et al.* A comprehensive genome-scale model for *Rhodosporidium toruloides* IFO0880 accounting for functional genomics and phenotypic data. *Metab. Eng. Commun.* **9,** e00101 (2019).
6. Zomorrodi, A. R. & Maranas, C. D. Improving the iMM904 S. cerevisiae metabolic model using essentiality and synthetic lethality data. *BMC Syst. Biol.* **4,** 178 (2010).
7. Xu, F., Gage, D. & Zhan, J. Efficient production of indigoidine in *Escherichia coli*. *J. Ind. Microbiol. Biotechnol.* **42,** 1149–1155 (2015).
8. Wehrs, M. *et al.* Sustainable bioproduction of the blue pigment indigoidine: Expanding the range of heterologous products in *R. toruloides* to include non-ribosomal peptides. *Green Chem.* **21,** 3394–3406 (2019).
9. Wehrs, M. *et al.* Correction: Sustainable bioproduction of the blue pigment indigoidine: Expanding the range of heterologous products in *R. toruloides* to include non-ribosomal peptides. *Green Chem.* (2019). doi:10.1039/C9GC90091H
10. Wehrs, M. *et al.* Production efficiency of the bacterial non-ribosomal peptide indigoidine

relies on the respiratory metabolic state in *S. cerevisiae*. *Microb. Cell Fact.* **17,** 193 (2018).

11. Burgard, A. P., Pharkya, P. & Maranas, C. D. Optknock: a bilevel programming framework for identifying gene knockout strategies for microbial strain optimization. *Biotechnol. Bioeng.* **84,** 647–657 (2003).
12. Nieto, C., Fernández-Tresguerres, E., Sánchez, N., Vicente, M. & Díaz, R. Cloning vectors, derived from a naturally occurring plasmid of *Pseudomonas savastanoi*, specifically tailored for genetic manipulations in *Pseudomonas*. *Gene* **87,** 145–149 (1990).
13. Pfeifer, B. A., Admiraal, S. J., Gramajo, H., Cane, D. E. & Khosla, C. Biosynthesis of complex polyketides in a metabolically engineered strain of *E. coli*. *Science* **291,** 1790–1792 (2001).
14. Heirendt, L. *et al.* Creation and analysis of biochemical constraint-based models using the COBRA Toolbox v.3.0. *Nat. Protoc.* **14,** 639–702 (2019).
