# Supplementary material for: Genome-scale metabolic rewiring improves titers rates and yields of the non-native product indigoidine at scale
Source: Nat Commun. 2020 Oct 23;11:5385. doi: 10.1038/s41467-020-19171-4 (PMC7584609; doi:10.1038/s41467-020-19171-4)
Supplement: Supplementary file 3 — Reporting Summary [file 41467_2020_19171_MOESM3_ESM.pdf]

nature research

Corresponding author(s): Aindrila Mukhopadhyay, PhD

Last updated by author(s): September 24, 2020

Reporting Summary

Nature Research wishes to improve the reproducibility of the work that we publish. This form provides structure for consistency and transparency in reporting. For further information on Nature Research policies, see our Editorial Policies and the Editorial Policy Checklist.

Statistics

For all statistical analyses, confirm that the following items are present in the figure legend, table legend, main text, or Methods section.

n/a	Confirmed	
☐	☒	The exact sample size (*n*) for each experimental group/condition, given as a discrete number and unit of measurement
☐	☒	A statement on whether measurements were taken from distinct samples or whether the same sample was measured repeatedly
☐	☒	The statistical test(s) used AND whether they are one- or two-sided<br>*Only common tests should be described solely by name; describe more complex techniques in the Methods section.*
☒	☐	A description of all covariates tested
☒	☐	A description of any assumptions or corrections, such as tests of normality and adjustment for multiple comparisons
☐	☒	A full description of the statistical parameters including central tendency (e.g. means) or other basic estimates (e.g. regression coefficient) AND variation (e.g. standard deviation) or associated estimates of uncertainty (e.g. confidence intervals)
☐	☒	For null hypothesis testing, the test statistic (e.g. *F*, *t*, *r*) with confidence intervals, effect sizes, degrees of freedom and *P* value noted<br>*Give P values as exact values whenever suitable.*
☒	☐	For Bayesian analysis, information on the choice of priors and Markov chain Monte Carlo settings
☒	☐	For hierarchical and complex designs, identification of the appropriate level for tests and full reporting of outcomes
☒	☐	Estimates of effect sizes (e.g. Cohen's *d*, Pearson's *r*), indicating how they were calculated

Our web collection on statistics for biologists contains articles on many of the points above.

Software and code

Policy information about availability of computer code

Data collection	CellNetAnalyzer 2018.2, MATLAB 2017b, CPLEX 12.8, Gurobi Optimizer 8.1, Cobra Toolbox v.3.0. All custom code used in this study is available as Supplementary Data 4.
Data analysis	Geneious Prime (Differential gene expression analysis); GraphPad Prism (Generation of standard curve via linear regression; calculation of standard error and standard mean)

For manuscripts utilizing custom algorithms or software that are central to the research but not yet described in published literature, software must be made available to editors and reviewers. We strongly encourage code deposition in a community repository (e.g. GitHub). See the Nature Research guidelines for submitting code & software for further information.

Data

Policy information about availability of data

All manuscripts must include a data availability statement. This statement should provide the following information, where applicable:

- Accession codes, unique identifiers, or web links for publicly available datasets
- A list of figures that have associated raw data
- A description of any restrictions on data availability

The RNAseq datasets generated and/or analyzed during the current study are available available through NCBI-SRA associated with NCBI-Bioproject (Accession IDs: PRJNA580539 - PRJNA580574) and the DOE-JGI IMG database (Project ID: 505977). The proteomic datasets generated and/or analyzed during the current study are available through PanoramaWeb, accessible at https://panoramaweb.org/genome-scale-rewiring-indigoidine.url. All data generated in this manuscript are included in the data source file.

Field-specific reporting

Please select the one below that is the best fit for your research. If you are not sure, read the appropriate sections before making your selection.

☒ Life sciences ☐ Behavioural & social sciences ☐ Ecological, evolutionary & environmental sciences

For a reference copy of the document with all sections, see nature.com/documents/nr-reporting-summary-flat.pdf

Life sciences study design

All studies must disclose on these points even when the disclosure is negative.

Sample size	The number of samples was determined by the number of tubes or plates that could fit into a shaker platform. No statistical methods were used to pre-determine the sample size. For the quantification of indigoidine, samples were prepared and analyzed in at least biological triplicate. All experiments were additionally repeated at least one additional time again in biological triplicate. The control samples analyzed by proteomics were prepared in biological triplicate and the engineered strain was prepared with six biological replicates.
Data exclusions	No data was excluded from this study.
Replication	At least three independent biological replications were performed for each experiment. All replications were successful in the sense they were consistent with each other.
Randomization	Randomization of samples is not relevant or possible for differential RNA expression analysis where the genotypes of the samples must be known in order to process the material for RNA extraction. No other statistical comparisons were made in this study.
Blinding	Blinding of samples is not relevant or possible for differential RNA expression analysis where the genotypes of the samples must be known in order to process the material for RNA extraction. No other statistical comparisons were made in this study.

Reporting for specific materials, systems and methods

We require information from authors about some types of materials, experimental systems and methods used in many studies. Here, indicate whether each material, system or method listed is relevant to your study. If you are not sure if a list item applies to your research, read the appropriate section before selecting a response.

Materials & experimental systems

n/a	Involved in the study
☒	☐ Antibodies
☒	☐ Eukaryotic cell lines
☒	☐ Palaeontology and archaeology
☒	☐ Animals and other organisms
☒	☐ Human research participants
☒	☐ Clinical data
☒	☐ Dual use research of concern

Methods

n/a	Involved in the study
☒	☐ ChIP-seq
☒	☐ Flow cytometry
☒	☐ MRI-based neuroimaging
