# Supplementary material for: Genome-scale metabolic rewiring improves titers rates and yields of the non-native product indigoidine at scale
Source: Nat Commun. 2020 Oct 23;11:5385. doi: 10.1038/s41467-020-19171-4 (PMC7584609; doi:10.1038/s41467-020-19171-4)
Supplement: Supplementary file 4 — Description of Additional Supplementary Files [file 41467_2020_19171_MOESM4_ESM.pdf]

Description of Additional Supplementary Files

Supplementary Data 1

Gene Targets and Gene Sequences Used to Design the Synthetic CRISPR Interference gRNA Array.

Supplementary Data 2

Identification of essential genes in *P. putida* KT2440 using barcoded transposon mutagenesis (RB-TnSeq).

Supplementary Data 3

Additional gene targeting sets for growth coupled production of two additional target molecules using glucose as the starting carbon source in *P. putida.*

Supplementary Data 4

Specific reactions and customized codes used in this study.
