# Supplementary material for: Genome-scale metabolic rewiring improves titers rates and yields of the non-native product indigoidine at scale
Source: Nat Commun. 2020 Oct 23;11:5385. doi: 10.1038/s41467-020-19171-4 (PMC7584609; doi:10.1038/s41467-020-19171-4)
Supplement: Supplementary file 2 — Peer Review File [file 41467_2020_19171_MOESM2_ESM.pdf]

Editorial Note: This manuscript has been previously reviewed at another journal that is not operating a transparent peer review scheme. This document only contains reviewer comments and rebuttal letters for versions considered at *Nature Communications*. [Mentions of prior referee reports have been redacted.]

Reviewer #1: unavailable.

Reviewer #2 (Remarks to the Author):

Banerjee et al. describe an integrated workflow to achieve high product titers of a non-native compound indigoidine in P. putida KT2440. First, a duality-based mixed-integer optimization approach is used to compute constrained minimal cut sets (cMCS) to identify 14 growth-coupled interventions for product synthesis. In doing so, the authors also generated gene essentiality information for P. putida using a barcoded fitness library, which will serve as a valuable resource for future studies. Then, a multiplex CRISPRi is used to implement gene knockdowns to achieve a 0.33 gm indigoidine/gm glucose (~50% maximum theoretical yield) production level. Interestingly, the authors also noticed a switch in temporal indigoidine synthesis, from the stationary to the exponential phase. Overall, the work of Banerjee et al. is of sound scientific merit and is a nice contribution to the field. Some additional benchmarking studies may be needed to properly place this contributions within the current state of the art.

Major Comments

1. Similar to a previous reviewer, this reviewer also believes that this work belongs in a more specialized journal. The cMCS-based framework has been used previously by Harder et al., 2016, Metab. Eng. to produce a heterologous product (itaconic acid) in E. coli. The novelty of this work, thus, resides in 1) the 'omics guided workflow developed to filter solution sets, and 2) simultaneous knock-down of 14 gene targets.
The authors may want to compare their workflow with other popular tools (such as OptKnock etc.) and also integrate gene-protein-reaction relationships explicitly into the optimization framework (perhaps using an approach similar to OptORF), which could help filter-out solution sets. Furthermore, such a framework has been recently published as well (Schneider et al., 2020, PloS Comp Bio), and merits a comparison (perhaps in silico) with their developed protocol. However, the number of interventions levied is certainly highest till date, but confirmatory RNASeq by Banerjee et al. revealed that 9 out of the 14 interventions were indeed knocked-down, and 9 simultaneous editing events has been reported previously (Wijsman et al., 2019, FEMS Yeast Res).
2. Although the authors compute the maximum possible yield for other products (i.e., methyl ketone and arginine), the varying phenotypes for growth on galactose vs glucose questions the generalizability of the overall framework. It would be informative if the authors could demonstrate applicability of the developed framework on other organisms, such as R. toruloides (similar MTY as P. putida).
3. How did the authors ensure that the higher amounts of glutamine produced were channeled towards indigoidine instead of other metabolites featuring downstream of glutamate? Doing so in silico is trivial (equating to blocking the exchange/sink reactions for possible products), but how was this enforced in the experiments?

Minor Comments

1. L399-400: As glutamine is a part of the P. putida biomass equation, recommend rephrasing 'With the specifications used herein each calculated knockout strategy (cMCS) will ensure that growth is not feasible without biosynthesis of glutamine'.
2. L70: Recommend changing 'feasible even when cell grow is sub-optimal' to 'feasible even when cell growth is sub-optimal'.
3. Supplementary Table 1 omits that DMALRED is also encoded by PP_0751, PP_1251, and PP_2925.

Reviewer #3 (Remarks to the Author):

The authors addressed my major points very well and satisfactorily. I have only few remaining minor issues after which the manuscript should be ready for publication:

1) It is a matter of taste, but I find the commas before and after indigoidine in the title somewhat odd as they disturb the flow of reading. The authors might therefore consider rewording the title. Actually, I think it would still be correct to just leave the two commas away?!

2) In their described MCS calculation and evaluation algorithm the authors manually converted the reaction MCS to gene MCS. Meanwhile, an extended algorithm for MCS calculation has been published (Schneider et al., PLOS Comp Biol, 2020) that allows direct calculation of gene MCS and should therefore be cited as a relevant development (e.g. in the Discussion section): https://doi.org/10.1371/journal.pcbi.1008110

3) Line 71: grow◊growth

4) Line 303: 1,3-BDO ◊ 1,4-BDO

5) Line 312/313: “It allows production while minimizing growth …” ◊ “It demands production of the target metabolite (e.g. to generate ATP for non-growth associated maintenance processes) even when cells do not grow …”

6) Line 403: you certainly did not compute ALL minimal combinations (MCS) of reaction knockouts blocking undesired fluxes. As you used it, the algorithm rather delivers only a (more or less random) selection of these MCS. You should simply remove the word “ALL”.

REVIEWER COMMENTS

Reviewer #2 (Remarks to the Author):

Banerjee et al. describe an integrated workflow to achieve high product titers of a non-native compound indigoidine in P. putida KT2440. First, a duality-based mixed-integer optimization approach is used to compute constrained minimal cut sets (cMCS) to identify 14 growth-coupled interventions for product synthesis. In doing so, the authors also generated gene essentiality information for P. putida using a barcoded fitness library, which will serve as a valuable resource for future studies. Then, a multiplex CRISPRi is used to implement gene knockdowns to achieve a 0.33 gm indigoidine/gm glucose (~50% maximum theoretical yield) production level. Interestingly, the authors also noticed a switch in temporal indigoidine synthesis, from the stationary to the exponential phase. Overall, the work of Banerjee et al. is of sound scientific merit and is a nice contribution to the field. Some additional benchmarking studies may be needed to properly place this contributions within the current state of the art.

We thank the reviewer for their overall positive assessment of our revised manuscript. We have added several new paragraphs to the discussion section to compare our work with recent advances in the field. This addition is specifically addressed in lines 297-330, and goes into detail regarding our choice of MCS optimization over other commonly used programs for our workflow. The new sections were guided by the reviewer's comment to compare workflows (Major Comment 1, second section). As suggested later in this referee report by Reviewer 1, we also show output from Optknock used to calculate solution sets for indigoidine production from glucose. Several references suggested by both reviewers have also been incorporated, as detailed below.

Major Comments

1. Similar to a previous reviewer, this reviewer also believes that this work belongs in a more specialized journal. The cMCS-based framework has been used previously by Harder et al., 2016, Metab. Eng. to produce a heterologous product (itaconic acid) in E. coli. The novelty of this work, thus, resides in 1) the 'omics guided workflow developed to filter solution sets, and 2) simultaneous knock-down of 14 gene targets.

Nature Communications is an ideal journal to reach bioengineering and biotechnologists interested in using genome scale metabolic models, which are increasingly being used in synthetic biology. The framework described here is a generally applicable template/ prototype to use computationally driven, genome scale metabolic model based

prediction in conjunction with a multiplexed CRISPRi dCpf1/dCas12 for experimental prototyping and validation.

Many aspects from our study could be of interest to a broad audience. We demonstrate both the use of heterologous non-native carbon streams (galactose) as well as the expression of heterologous gene pathways. Both were tested and optimized using the workflow we described. We emphasize that this is the first report where the production phenotype has shifted from stationary to exponential phase, as we saw in under fed-batch cultivation in a bioreactor. The ensemble tools used here can be used in both eukaryotes and prokaryotes; the rate limiting step for adoption is generation and transformation of synthetic DNA.

As the reviewer has correctly described, a cMCS based framework has been previously used by Harder *et al*, 2016. In the previous review round, Reviewer 3 had also identified Harder *et al* as important work in the field, and we have cited this work in the introduction (line 64). In contrast to our study, Harder *et al* used core metabolism to compute MCS and the final engineered strain was a glutamate auxotroph. Their study is valuable, but would be challenging to extend the study to use a genome scale model for growth coupled strain design because it was focussed to enumerate smallest cMCS rather than the ones that would result in growth coupled metabolite production. In this review round, we have also clarified the first line of the discussion to read as follows:

Revised line 287:

> This study is the first implementation of genome scale MCS predictions enabled with CRISPR interference that resulted in a strain where production was paired with growth

to better distinguish this work from Harder *et al.*

The authors may want to compare their workflow with other popular tools (such as OptKnock etc.) and also integrate gene-protein-reaction relationships explicitly into the optimization framework (perhaps using an approach similar to OptORF), which could help filter-out solution sets. Furthermore, such a framework has been recently published as well (Schneider et al., 2020, PloS Comp Bio), and merits a comparison (perhaps in silico) with their developed protocol.

We agree with the reviewer's comment, as the suggested approach helps contextualize our work with other approaches in the field. We have revised the discussion to help the reader understand the rationale between different optimization algorithms in a new paragraph (lines 297-319). Our workflow is agnostic to the specific algorithm used to generate reaction targets - output from any of these computational methods can be

readily evaluated for miscategorized/underrepresented essential genes and gRNAs targeting genes for knockdown can be synthesized. These optimization strategies make different assumptions on cellular metabolism and have been extensively reviewed elsewhere (Alter and Ebert 2019, BMC Bioinfo.; Maia et al. 2016, BMC Bioinfo.; Machado and Herrgård 2015, Metab. Eng. Comm.), and we now include these references in the discussion (line 304).

To directly address the reviewer's comment, we now include a solution set for the production of glutamine from glucose using OptKnock (Supplementary Table 5) and include additional sentences suggesting the scope of other algorithms in conjunction with our overall process (line 311-314). These solutions might be improved by using modifications in the OptKnock algorithm (tilted objective, OptKnock in combination with other Opt- approaches, etc.) which is beyond the scope of this study.

New section in manuscript (lines 297-319):

> In contrast, The MCS algorithm does not maximize growth and is unbiased towards any part of the solution space. Further, the MCS algorithm allows us to set boundaries for minimum demanded production and growth, thus providing strong coupling constrained MCS (cMCS) solution sets. These optimization strategies make different assumptions on cellular metabolism and have been extensively reviewed elsewhere[43–45]. OptKnock related methods have been used in growth coupled strategies for primary alcohols (1,4-BDO[46]) and organic acids (succinate[47], lactate[48]). However, for a large subset of native metabolites including amino acids (serine, glutamate, glutamine), FBA-based approaches often result in "non unique" solutions, which are optimized for production but not necessarily for growth. In contrast, the recently delineated MCS-based approach[8] provides strong coupled solutions sets for a large number of metabolites within a reasonable computation time and fit well with the downstream biological constraints for CRISPR/dCpf1 rewiring. A comparison of solution sets generated with OptKnock (**Supplementary Table 5**), versus that with cMCS (using similar computation time, targetable reactions, number of reactions to be deleted, etc.), did not provide strong coupled strategies although several "non unique" solutions exist. Other studies have described growth coupling as the creation of a "driving force" such as ATP production or cofactor imbalance, and link the driving force to the desired production pathway[41,49–51]. Driving force coupling is also pathway specific and requires additional strain engineering. Examples include 1-butanol production in *E. coli* using NADH as the driving force[49] or media supplementation for butanone production in *E. coli* linked to acetate assimilation[41].

Next, we thank the reviewer for mentioning Schneider *et al* 2020, which was published while this paper was under review. In the pipeline described in our manuscript, we filter out solution sets based gene-protein reaction relationships (GPRs) that translate to an experimentally feasible gene deletion set. This includes several criteria as shown in Figure 1a and Supplementary Table 3. The solution sets are first computed using the

cMCS algorithm from Kamp and Klamt, 2017. The work described in Schneider *et al*. 2020 extends their earlier published cMCS algorithm to compress and integrate GPRs in an automated workflow which occurs before MCS are computed. We now reference Schneider *et al* in the discussion as it will simplify the use of GSMM in our workflow (lines 328-330). The framework from Schneider et al. is already being implemented in our laboratory for new inquiries in strain engineering.

New text in manuscript (line 328-330):

> Most recently, the MCS algorithm was extended to integrate GPRs before the cMCS are computed[54] that would streamline an inefficient step in our workflow.

New Supplementary Table

> **Supplementary Table 5:** Comparison of output from OptKnock and cMCS algorithms to identify gene cut sets to improve indigoidine yield through growth coupling to alpha-ketoglutarate or glutamine.

Growth coupling for: alpha-ketoglutarate		
Algorithm	OptKnock	cMCS
Number of reactions for knockout	4	21
Reactions	GAPD/PGK, SUCDi, TALA/TKT1, TPI	ABTA, AGM3PA, AGM3PH, AGM4PA, AGM4PH, AKGDH, ALDD2x, DHORD2, FDH, GAD2ktpp, GLCNtex, LDH_D, LSERDHr, PDH, PDHcr, RBK, SUCD4, SUCDi, TALA, GLCabcpp/HEX1, MCITD/MCITL2/MCITS/MICITDr
YPS[a]	0.056 to 0.473	1.085
YBS	0.012	0.0138
Growth coupling for: glutamine		
Algorithm	OptKnock	cMCS
Number of reactions for knockout	15	15

rxns	ACOAD3f, ALLTN, ECOAH20, ECOAH4, FMNRx2, GLYCLTDy, ME2, NADH16pp, OAADC, PGI, PIt2rpp, PPA,PPCK, PPKr, TRPS3	ANHMK, D_LACt2pp, GLCDpp, H2CO3D, HCO3E, MDH, MDH2, ME2, NACODA, ORNDC ,PHAPC40, PPS, PROD2 ,TALA
YPS[a]	0 to 0.76	0.92
YBS	0.0105	0.0149
Indigoidine molar yields	<0.001	0.48

[a]YPS: Product yield (mole of product per mole of glucose)

However, the number of interventions levied is certainly highest till date, but confirmatory RNASeq by Banerjee et al. revealed that 9 out of the 14 interventions were indeed knocked-down, and 9 simultaneous editing events has been reported previously (Wijsman et al., 2019, FEMS Yeast Res).

Wijsman *et al* is a fine example of creative CRISPR/Cas9 engineering but its strength lies in leveraging specific traits in genome organization. Situations where a single gRNA targets multiple loci are usually avoided as promiscuous CRISPR/Cas9 (or Cpf1) binding events are considered deleterious off-target reactions. Owing to the high degree of sequence similarity between hexose transporters and fortuitous clustering of 6 transporters in gene proximal sequences, ORFs encoding 9 hexose transporters were deleted in a single transformation round by using gRNAs which could target multiple genetic loci. However, Wijsman and coworkers only needed to use 4 gRNAs and 8 repair templates in a given strain-editing round. A total of 21 transporters were deleted after 3 sequential rounds of transformation. The authors constructed a small gRNA plasmid library where each plasmid contained 2 gRNAs, whereas we constructed a single plasmid containing a 14 gRNA array. In contrast to our study, we targeted 14 single-copy genetic loci and did not need to generate linear repair templates. Subsequent validation by RNAseq confirmed that nine of the targeted genes were conditionally knocked down. Regardless, we now cite this work in the discussion (Lines 360-362) as an example of multiplex CRISPR strain engineering relevant for building deletion strains. We also have clarified that all genes identified for inactivation are single copy and at dispersed locations throughout the genome (lines 140-142).

New lines added to manuscript (lines 140-142):

Eight of these 14 reactions are present in central metabolism and when mapped to their corresponding genes and gene products, represent 16 single-copy genes dispersed throughout the genome (**Figure 1b** and **Supplementary Dataset 1**).

New citation added to manuscript (lines 360-362, reference 56):

The plasmid based CRISPRi system retained stable phenotype for six days, but can be further stabilised using genomic integration of the dCpf1/CRISPRi system or by developing multiplex gene deletion strategies[55,56].

2. Although the authors compute the maximum possible yield for other products (i.e., methyl ketone and arginine), the varying phenotypes for growth on galactose vs glucose questions the generalizability of the overall framework. It would be informative if the authors could demonstrate applicability of the developed framework on other organisms, such as R. toruloides (similar MTY as P. putida).

The reviewer is concerned regarding the generalizability of the work because the production from galactose is not as consistent as production with glucose. We explain this difference in variability from glucose in that galactose is a non-native substrate and we engineered the strains to consume galactose using a heterologous galactose catabolism pathway gene pathway from *E. coli* (*galETKM*). Several lines in the results section were rewritten to clarify that we mined the *E. coli* literature to identify known gene pathways that could enable the strain engineering work in *P. putida* necessary to use this carbon source (lines 164-165). As we described in the methods section (lines 563-565), the initial adaptation of *P. putida* KT2440 *Ec_galETKM* requires a reproducible 4-5 day lag phase from the initial dilution in LB media to M9 media supplemented with 1% galactose. Additional work using adaptive lab evolution (manuscript in preparation) has identified the genetic basis required for a reduced lag phase and increased growth rates using this non-native carbon source. Additionally, characterizing metabolic flux with 13C MFA analysis in these evolved strains can help clarify why differences in galactose catabolism compared to glucose catabolism. To address the reviewer's concern here, we have softened the language around describing the production values as consistent by explicitly referring to the use of the native carbon source (glucose) in the discussion (lines 353-355).

New sentence in manuscript (lines 164-165):

While *P. putida* cannot natively consume galactose, the galactose catabolic pathway has been well characterized in E. coli[25,26].

Revised line 353-355:

Even with the limitations described above, our approach also allowed us to achieve, in one cycle of strain engineering, a high and consistent TRY for indigoidine from glucose across cultivation scales.

While the reviewer's suggestion to implement our workflow in *Rhodosporidium toruloides* is a tantalizing idea, it is unfortunately out of scope of this work. We would need to optimize many tools being developed by our collaborators for use in our laboratory (characterize and optimize equivalent minimal salt culture media for product yield calculations; convert Cas9 or Cas12 CRISPR systems to CRISPRi systems; build methods to characterize essential genes in this organism; evaluate plasmid/linear DNA transformation methods, and so forth).

3. How did the authors ensure that the higher amounts of glutamine produced were channeled towards indigoidine instead of other metabolites featuring downstream of glutamate? Doing so in silico is trivial (equating to blocking the exchange/sink reactions for possible products), but how was this enforced in the experiments?

We thank the reviewer for this question about carbon flux being diverted towards other possible products. Experimentally, the main steps taken that enhance channeling of glutamine to indigoidine are part of the multi-gene metabolic rewiring. Specifically, three central metabolic pathway genes which convert glutamate to other metabolites were targeted for inhibition in this rewiring strategy (PP_4947/*putA*; PP_0864/*speC*; and PP_5186/*argE*), as diagrammed in Supplementary Figure 1. By proteomics and RNAseq, we were able to validate that both *putA* and *speC* were inhibited, but were unable to confirm that *argE* RNA or protein levels had decreased. As we describe in the discussion, further improvements to knockdown efficiency may improve the final product titer in our strains (lines 360-362). Current levels of indigoidine produced in this engineered strain (highest titers/yields: 25.6 g/L, 0.33 g indigoidine per g of glucose) represents greater than half of maximum glutamine possible. Thus we estimate that the majority of the glutamine is being channelled to indigoidine. However, glutamine remains available for anabolic processes and is used in other steps (via glutamate, as pointed out by the reviewer).

The model allowed for 10% biomass accumulation and in our cultivations, the engineered strain remains a glutamine prototroph. We did not detect any additional secreted extracellular products or overflow metabolites in the supernatant (i.e. gluconate, ketogluconate, lactate, acetate) that could be excreted by *P. putida*. Further, we also see no increase in biomass relative to WT. In this scenario, less than 4% of total intracellular glutamine would be sufficient for maintaining the biomass observed.

Specifically, based on the biomass equation formulation used in the genome scale model, the biomass yield for the engineered strain would account for about 3.3% glutamine and 4% (glutamate equivalents) accumulated. In *P. putida* central metabolism, glutamate can be converted into alpha-ketoglutarate, fumarate, aspartate, proline, ornithine, and glutamine. These characterized pathways result in flux redistribution towards alpha ketoglutarate, fumarate or aspartate, in addition to the pathways that are targeted as shown in Supplementary Figure 1. The first two metabolites (alpha ketoglutarate, fumarate) redirect flux toward TCA whereas biosynthesis of aspartate would require equimolar amounts of oxaloacetate. Targeting the remaining reactions downstream of glutamate for inactivation would have a negligible impact on predicted indigoidine titer. Finally, conversion to indigoidine is highly efficient via the *sfp/ bpsA* pathway, using glutamine as the sole precursor (Pang et al, J. Am. Chem. Soc. 2020), and has been used as an assay for measuring glutamine (WIPO Patent Application WO2015084189A1). We essentially relied on the known activity of the pathway for efficient conversion of glutamine to indigoidine, though this too could be improved further. All these are important considerations, especially when considering combinatorial approaches in the future, and we thank the reviewer for pointing this out and have now added several sentences to discuss this (lines 364-365, new Supplementary Discussion).

New sentence, (lines 364-365):

> Additional reduction of competing reactions that draw on glutamate might only have a negligible impact on predicted indigoidine titer (Supplementary Discussion).

New Supplementary Discussion in Supplementary File:

> In *P. putida* central metabolism, glutamate can be converted into alpha-ketoglutarate, fumarate, aspartate, proline, ornithine, and glutamine. These characterized pathways result in flux redistribution towards alpha ketoglutarate, fumarate or aspartate, in addition to the pathways that are targeted as shown in **Supplementary Figure 1**. The first two metabolites (alpha ketoglutarate, fumarate) redirect flux toward TCA whereas biosynthesis of aspartate would require equimolar amounts of oxaloacetate. If the biomass yields are the same as that of WT, based on biomass equation formulation used in the genome scale model, in the engineered strain about 3.3% glutamine and 4% (glutamate equivalents) accumulated intracellularly would be sufficient for 10% biomass yield. Using the experimentally observed indigoidine yields in WT and engineered *P. putida* strains, we find that only about 10% of MTY glutamine in WT is converted to indigoidine whereas 70% of the MTY glutamine was successfully converted to indigoidine in the engineered strain. The set of exchange reactions that might in silico impact growth coupled indigoidine production in the engineered strain are for pyruvate, alpha ketoglutarate, leucine, valine, citrate, isocitrate and D-alanine. Biosynthesis of all these metabolites are upstream of glutamate. Our HPLC method did not detect any organic acids. Of

> the remaining three amino acids, leucine, valine and D-alanine are synthesized from pyruvate. Blocking these pathways above would generate auxotrophies for the indicated metabolites.

Minor Comments

1. L399-400: As glutamine is a part of the P. putida biomass equation, recommend rephrasing 'With the specifications used herein each calculated knockout strategy (cMCS) will ensure that growth is not feasible without biosynthesis of glutamine'.

Both Reviewer 2 and Reviewer 3 identified that this sentence could be clarified. We have rephrased the sentence to the following:

> With the specifications used herein each calculated knockout strategy (cMCS) demands production of glutamine even when cells do not grow.

2. L70: Recommend changing 'feasible even when cell grow is sub-optimal' to 'feasible even when cell growth is sub-optimal'.

We have modified the text as suggested.

3. Supplementary Table 1 omits that DMALRED is also encoded by PP_0751, PP_1251, and PP_2925.

We thank the reviewer for catching this oversight. We have included this information in Supplementary Dataset 1 (Supplementary Table 1 describes potential growth coupled metabolites between *E. coli* and *P. putida.*)

Reviewer #3 (Remarks to the Author):

The authors addressed my major points very well and satisfactorily. I have only few remaining minor issues after which the manuscript should be ready for publication:

We are glad that we were able to address your concerns and as a result believe the manuscript has improved significantly through the review process.

1) It is a matter of taste, but I find the commas before and after indigoidine in the title somewhat odd as they disturb the flow of reading. The authors might therefore consider rewording the title. Actually, I think it would still be correct to just leave the two commas away?!

We have modified the title as suggested.

2) In their described MCS calculation and evaluation algorithm the authors manually converted the reaction MCS to gene MCS. Meanwhile, an extended algorithm for MCS calculation has been published (Schneider et al., PLOS Comp Biol, 2020) that allows direct calculation of gene MCS and should therefore be cited as a relevant development (e.g. in the Discussion section):
https://doi.org/10.1371/journal.pcbi.1008110

Reviewer 2 has also mentioned this recently published work (Schneider *et al*) and we agree it is valuable to include. We now cite this work in the discussion (Lines 328-330).

3) Line 71: grow with growth

Fixed as suggested.

4) Line 303: 1,3-BDO with 1,4-BDO

We thank the reviewer for catching this typo. It has been corrected throughout the manuscript.

5) Line 312/313: “It allows production while minimizing growth …” with “It demands production of the target metabolite (e.g. to generate ATP for non-growth associated maintenance processes) even when cells do not grow …”

We have made this change as suggested.

6) Line 403: you certainly did not compute ALL minimal combinations (MCS) of reaction knockouts blocking undesired fluxes. As you used it, the algorithm rather delivers only a (more or less random) selection of these MCS. You should simply remove the word “ALL”.

Corrected as suggested. We thank the reviewers for their careful and thoughtful read of our manuscript and their insightful comments.

Reviewer #2 (Remarks to the Author):

I have no additional comments. The authors have adequately addressed all my comments and incorporated changes in the revision.

REVIEWERS' COMMENTS

Reviewer #2 (Remarks to the Author):

I have no additional comments. The authors have adequately addressed all my comments and incorporated changes in the revision.

We thank the reviewer for their thoughtful consideration of the manuscript.
